# Direct C–H amination reactions of arenes with *N*-hydroxyphthalimides catalyzed by cuprous bromide

**DOI:** 10.3762/bjoc.18.65

**Published:** 2022-06-03

**Authors:** Dongming Zhang, Bin Lv, Pan Gao, Xiaodong Jia, Yu Yuan

**Affiliations:** 1 College of Chemistry and Chemical Engineering, Yangzhou University, Yangzhou 225002, P. R. of China

**Keywords:** amination, copper, *N*-hydroxyphthalimides, radical reactions, triethyl phosphite

## Abstract

An efficient Cu-catalyzed strategy for the direct C–H amination of arenes in high yields using *N*-hydroxyphthalimide as the amidyl radical precursor under air is reported. A possible mechanism is proposed that proceeds via a radical reaction in the presence of CuBr and triethyl phosphite.

## Introduction

Practical methods for constructing C–N bonds are in high demand in organic synthesis since nitrogen-containing organic compounds are widely used in biologically active substances [1], multifunctional materials [2–3], and metal ligands [4–5]. Among them, the synthesis of aromatic amines has been important to researchers in recent decades. With the combination of C–H activation, many aminations of aryl compounds have been established [6–16].

**Scheme 1:**
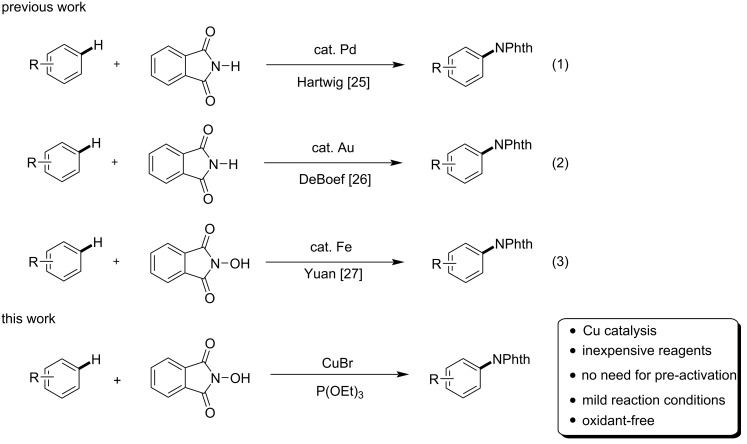
Amination of arenes with phthalimides.

However, it is necessary to introduce the directing group into the arene in most successful cases. As a good amino source, phthalimides have been widely applied in amination reactions [17–24]. Especially, *N*-hydroxyphthalimide can react with arenes directly in the presence of palladium [25] or gold [26] (Scheme 1, reactions 1 and 2). Recently, we found that iron catalyzes the amination of arenes with *N*-hydroxyphthalimide under air (reaction 3) [27]. Herein, we report a method for the construction of aromatic amines via the copper-catalyzed intermolecular radical amination of arenes with *N*-hydroxyphthalimide (NHPI) under air.

## Results and Discussion

Initially, *N*-hydroxyphthalimide (NHPI, **2a**) was reacted with benzene, catalyzed by CuBr (40 mol %) in the presence of P(OEt)_3_ (6 equiv, triethyl phosphite) under air at 100 °C (Table 1). The yield of the corresponding amide **3a** was 78% (Table 1, entry 1). The reaction was completely inhibited in the absence of the copper catalyst or P(OEt)_3_, and no product was detected (Table 1, entries 2 and 3). Different copper salts were tested and the reactions proved to be less efficient (Table 1, entries 4 and 5). Except for triethyl phosphite, the reaction could not be carried out with other phosphorus species (Table 1, entries 6–8). The optimum result was obtained when benzene was employed as the substrate and solvent without additional solvent (Table 1, entries 9 and 10). It was found that a higher or lower temperature decreased the yield of the reaction (Table 1, entries 12 and 13). Meanwhile, the product yield was not increased by prolonging the reaction time from 12 to 24 hours (Table 1, entry 14). The yield of the product was a bit lower (55%) when the reaction was operated under argon atmosphere instead of air (Table 1, entry 15).

**Table 1:** Optimization of the reaction conditions.^a^

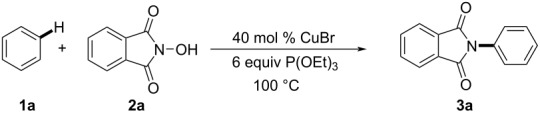					

Entry	Catalyst	Additive	Temp (°C)	Solvent	Yield (%)^b^

1	CuBr	P(OEt)_3_	100	–	78
2	none	P(OEt)_3_	100	–	NR
3	CuBr	none	100	–	NR
4	Cu(MeCN)_4_PF_6_	P(OEt)_3_	100	–	15
5	CuBr_2_	P(OEt)_3_	100	–	33
6	CuBr	P(OMe)_3_	100	–	55
7	CuBr	P(O)(OMe)_3_	100	–	trace
8	CuBr	P(*t-*Bu)_3_	100	–	trace
9	CuBr	P(OEt)_3_	100	MeCN	72
10	CuBr	P(OEt)_3_	100	DCE	65
11^c^	CuBr	P(OEt)_3_	100	–	40
12	CuBr	P(OEt)_3_	70	–	61
13	CuBr	P(OEt)_3_	120	–	70
14^d^	CuBr	P(OEt)_3_	100	–	77
15^e^	CuBr	P(OEt)_3_	100	–	55

^a^Reaction conditions: **1a** (2.0 mL as substrate and solvent), **2a** (0.10 mmol), CuBr (0.04 mmol) and P(OEt)_3_ (0.6 mmol) were stirred for 12 h at 100 °C under air. ^b^Isolated yield after chromatography. ^c^P(OEt)_3_ (0.2 mmol) was added. ^d^Reaction time: 24 h. ^e^Under argon atmosphere.

**Scheme 2:**
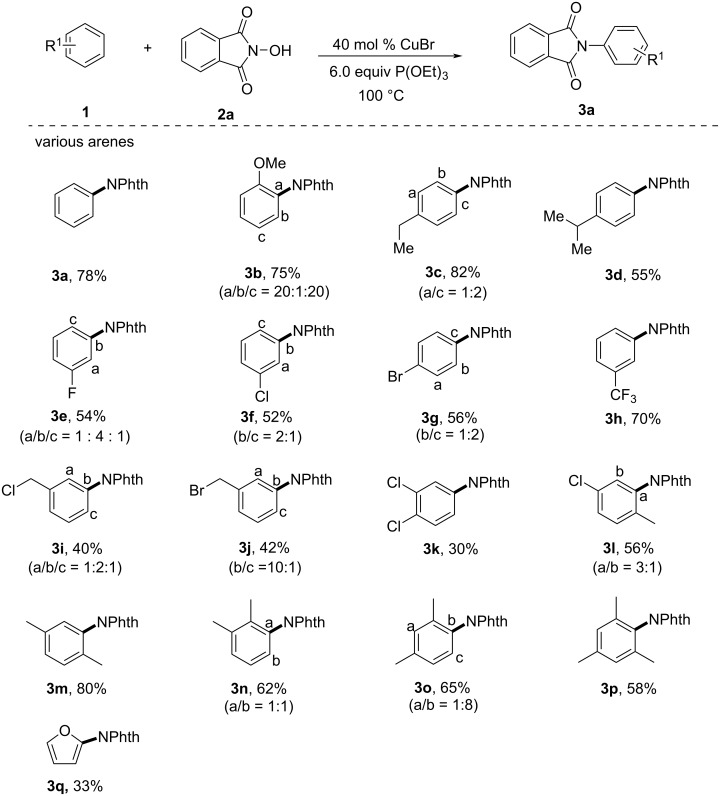
Substrate scope of the copper-catalyzed C–H imidation of arenes. Reaction conditions: **1** (2.0 mL as substrate and solvent), **2a** (0.10 mmol), CuBr (0.04 mmol) and P(OEt)_3_ (0.6 mmol) were stirred for 12 h at 100 °C under air. a = *ortho*-, b = *meta*-, c = *para*-.

**Scheme 3:**
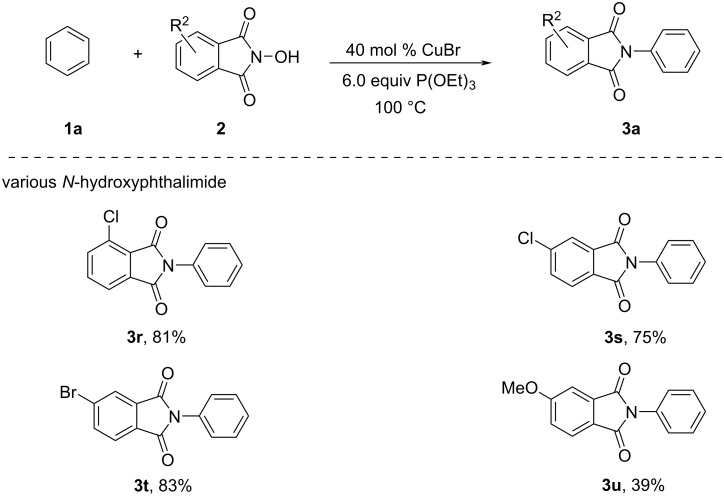
Substrate scope of the copper-catalyzed C–H imidation of *N*-hydroxyphthalimide. Reaction conditions: **1a** (2.0 mL as substrate and solvent), **2** (0.10 mmol), CuBr (0.04 mmol) and P(OEt)_3_ (0.6 mmol) were stirred for 12 h at 100 °C under air.

Next, we investigated the phthalimidation of various arenes **1** with *N*-hydroxyphthalimides **2** under the optimized conditions (Scheme 2). All the arenes tested, including mono-, di-, and tri-substituted aromatics and furans, reacted with *N*-hydroxyphthalimide (**2a**) smoothly to afford good yields of the corresponding products. Anisole was well tolerated in this reaction and gave the phthalimide product **3b** in 75% yield, mainly as *ortho-* and *para*-substituted products. Electron-donating alkyl-substituted arenes were generally more prone to give *ortho* and *para* products. When the reaction was carried out with cumene under standard conditions, due to steric hindrance resulting from the alkyl group on the arene’s ring, only the single product **3d** was observed in 55% yield. At the same time, arenes containing electron-withdrawing groups (such as halogen and trifluoromethyl) also give the corresponding products **3e–h** in moderate yields with the *meta* substituted isomers as the major products. Interestingly, only the *meta* product was detected when (trifluoromethyl)benzene was treated with **2a**. Moreover, benzyl chloride and benzyl bromide tolerated the reaction conditions, affording the aryl-phthalimidated products of which the *ortho* and *para* products were the major isomers. It is also shown that disubstituted and trisubstituted arenes successfully reacted to give the corresponding products in good yields. In addition, employing furan as the substrate, imidation occurred only at the *ortho* position to provide **3q** with a moderate 33% yield. Finally, various substituted *N*-hydroxyphthalimides were studied (Scheme 3), and the desired *N*-phthalimide products **3r–u** were obtained in moderate to high yields.

**Scheme 4:**
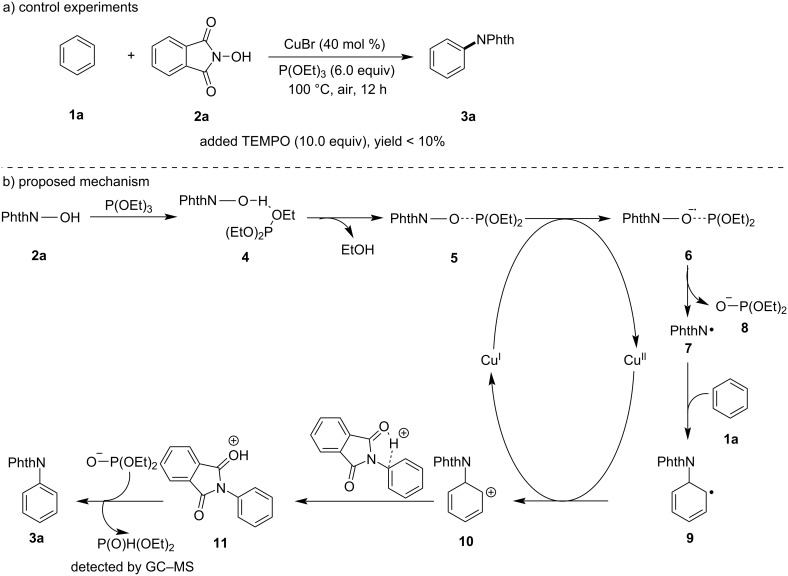
A plausible reaction mechanism.

According to the experimental results and our previous work [27–28], a possible reaction mechanism is given in Scheme 4. At first, NHPI combines with triethyl phosphite to form intermediate **4**, which is the loss of ethanol to generate intermediate **5**. Then, single-electron transfer (SET) between CuBr and intermediate **5** forms intermediate **6**, which initiates the N–O bond homolytic cleavage resulting in forming an *N*-centred phthalimidyl radical **7** (PhthN•) and anion **8**. Meanwhile, Cu(I) is oxidized to Cu(II) in this step. Next, radical **7** attacks the benzene via radical addition to generate the intermediate **9**, which is oxidized by Cu(II) to give **10**, which undergoes aromatization and deprotonation to afford the product **3a**. At this stage, Cu(I) is regenerated to complete the catalytic cycle.

## Conclusion

In summary, we have developed a convenient copper-catalyzed method for the direct C–H amination of arenes in good yields using *N*-hydroxyphthalimides as the amido radical precursor under mild conditions. This reaction has a broad substrate scope and leads to moderate to good yields in most cases. Also, good chemoselectivities were observed with some substrates. It is envisaged that this work will provide a simple amination strategy for synthesizing aromatic amines.

## Experimental

All new compounds were fully characterized. ^1^H NMR and ^13^C NMR spectra were obtained with Agilent Technologies AVANCE-400 MHz or 600 MHz spectrometers in CDCl_3_ as the solvent with TMS as an internal standard. Mass spectra were obtained on a Bruker Dalton maXis instrument. All reactions were carried out under air. Unless otherwise noted, materials were obtained from commercial suppliers and were used without further purification. All reactions under standard conditions were monitored by thin-layer chromatography (TLC) on gel F254 plates. Flash column chromatograph was carried out using 300–400 mesh silica gel at medium pressure.

**General procedure for synthesis of 3a–u: ***N*-Hydroxyphthalimide (0.1 mmol), CuBr (40 mol %, 0.04 mmol), triethyl phosphite (6.0 equiv, 0.6 mmol) and (hetero)arene (2 mL) were added into a 15 mL sealed tube. The resulting mixture was stirred at 100 °C under air for 12 h, and the progress was monitored by TLC. The solution was then cooled to room temperature and the solvent was removed under vacuum. The crude residue was purified by column chromatography on silica gel (ethyl acetate/petroleum ether 1:10) to afford the desired products **3a–u**.

## Supporting Information

File 1 Synthetic schemes for products, characterization data, and copies of ^1^H, ^13^C, and ^19^F NMR spectra.
