# Supplementary material for: Direct C–H amination reactions of arenes with N-hydroxyphthalimides catalyzed by cuprous bromide
Source: Beilstein J Org Chem. 2022 Jun 3;18:647–52. doi: 10.3762/bjoc.18.65 (PMC9174840; doi:10.3762/bjoc.18.65)
Supplement: File 1 — Synthetic schemes for products, characterization data, and copies of 1H, 13C, and 19F NMR spectra. [file Beilstein_J_Org_Chem-18-647-s001.pdf]

BEILSTEIN JOURNAL OF ORGANIC CHEMISTRY

Supporting Information

for

Direct C–H amination reactions of arenes with *N*-hydroxyphthalimides catalyzed by cuprous bromide

Dongming Zhang, Bin Lv, Pan Gao, Xiaodong Jia and Yu Yuan

Beilstein J. Org. Chem. **2022,** *18,* 647–652. doi:10.3762/bjoc.18.65

Synthetic schemes for products, characterization data, and copies of ^{1}H, ^{13}C, and ^{19}F NMR spectra

License and Terms: This is a supporting information file under the terms of the Creative Commons Attribution License (https://creativecommons.org/licenses/by/4.0). Please note that the reuse, redistribution and reproduction in particular requires that the author(s) and source are credited and that individual graphics may be subject to special legal provisions.
The license is subject to the *Beilstein Journal of Organic Chemistry* terms and conditions: (https://www.beilstein-journals.org/bjoc/terms)

1. General information

All new compounds were fully characterized. ^{1}H NMR and ^{13}C NMR spectra were obtained with Agilent Technologies AVANCE-400MHz or 600MHz spectrometers in $CDCl_3$ as the solvent and with TMS as an internal standard. Mass spectra were obtained on a Bruker Dalton maXis instrument. All reactions were carried out under air. Unless otherwise noted, materials were obtained from commercial suppliers and were used without further purification. All reactions under standard conditions were monitored by thin–layer chromatography (TLC) on gel F254 plates. Flash column chromatograph was carried out using 300–400 mesh silica gel at medium pressure.

2. Arylamines general procedure

N-hydroxyphthalimide (0.1 mmol), CuBr (40 mol %, 0.04 mmol), triethyl phosphite (6.0 equiv, 0.6 mmol) and (hetero)arene (2 mL) were added to a 15 mL sealed tube. The resulting mixture was stirred at 100 °C under air for 12 h and the progress monitored by TLC. The solution was then cooled to room temperature and the solvent was removed under vaccum. The crude residue was purified by column chromatography on silica gel (ethyl acetate/petroleum ether = 1:10) to afford the desired products **3a–u**.

3. Characterization of products

2-Phenylisoindoline-1,3-dione (3a)[1,2]

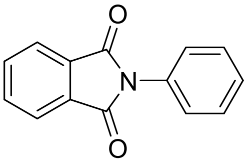

3a

Yield: 17.4 mg, (78%); white solid.

^{1}H NMR (400 MHz, CDCl$_3$): δ 7.96–7.94 (m, 2H), 7.82–7.75 (m, 2H), 7.53–7.49 (m, 2H), 7.47–7.35 (m, 3H).

^{13}C NMR (101 MHz, CDCl$_3$): δ 167.4, 134.5, 131.9, 131.8, 129.3, 128.3, 126.8, 123.9.

HRMS (ESI): m/z [M + Na]$^+$ calcd for $C_{14}H_9NNaO_2$: 246.0525; found: 246.0523.

2-(2-Methoxyphenyl)isoindoline-1,3-dione (3ba),

2-(3-methoxyphenyl)isoindoline-1,3-dione (3bb),

2-(4-methoxyphenyl)isoindoline-1,3-dione (3bc)[1,2]

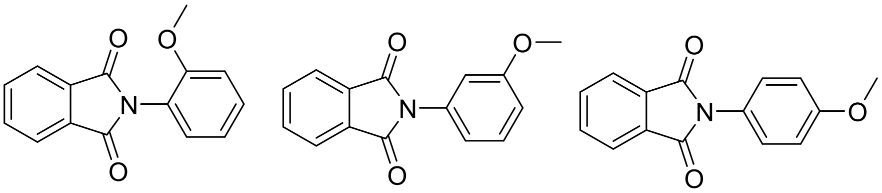

3ba **3bb** **3bc**

Yield: 18.9 mg, (75%); (**3ba**:**3bb**:**3bc** = 20:1:20); white solid. The ratio was determined by ^{1}H NMR spectroscopy.

^{1}H NMR (400 MHz, CDCl$_3$): δ 7.91–7.85 (m, 2H), 7.75–7.67 (m, 2H), 7.38 (td, *J* = 1.7 Hz, 1H [**3ba**], 1H [**3bb**]), 7.30–7.26 (m, 2H [**3bc**]), 7.22–7.19 (m, 1H [**3ba**]), 7.04–6.94 (m, 2H [**3ba**], 3H [**3bb**], 2H [**3bc**]), 3.78 (s, 3H [**3bc**]), 3.77 (s, 3H [**3bb**]), 3.74 (s, 3H [**3ba**]).

^{13}C NMR (101 MHz, CDCl$_3$): δ 167.7, 167.5, 159.3, 155.5, 134.4, 134.4 (2C), 134.2, 132.3, 131.9, 130.8, 130.1, 128.1, 123.8, 121.0, 114.6, 112.2, 55.9, 55.6.

HRMS (ESI): m/z [M + Na]$^+$ calcd for $C_{15}H_{11}NNaO_3$: 276.0631; found 276.0631.

2-(2-Ethylphenyl)isoindoline-1,3-dione (3ca),

2-(4-ethylphenyl)isoindoline-1,3-dione (3cb)[1,2]

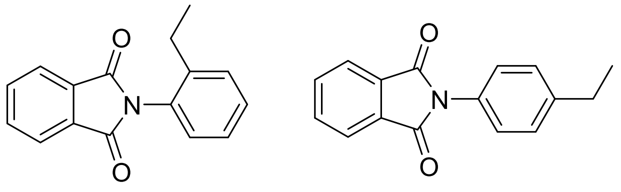

3ca **3cb**

Yield: 20.6 mg, (82%); (**3ca**:**3cb** = 1:2); white solid. The isomer ratio was determined by ^{1}H NMR spectroscopy.

^{1}H NMR (400 MHz, CDCl$_3$): δ 7.96–7.91 (m, 2H), 7.80–7.74 (m, 2H), 7.42 (t, 2H [**3ca**], 1H [**3cb**]), 7.32 (s, 1H [**3ca**]),7.26–7.20 (m, 3H [**3cb**]), 7.17 (d, *J* = 7.6 Hz, 1H [**3ca**]), 2.76–2.67 (m, 2H [**3cb**]), 2.52 (d, *J* = 7.6 Hz, 2H [**3ca**]), 1.26 (t, *J* = 7.6 Hz, 3H [**3cb**]), 1.16 (t, *J* = 7.6 Hz, 3H [**3ca**]).

^{13}C NMR (101 MHz, CDCl$_3$): δ 167.8, 145.5, 144.5, 134.5, 134.4, 132.1, 131.9 (2C), 129.9, 129.4, 129.1, 128.7, 127.9, 127.0, 126.6, 126.3, 124.1, 123.9, 123.8, 28.8, 24.5, 15.5, 14.4.

HRMS (ESI): m/z [M + Na]$^+$ calcd for $C_{16}H_{13}NNaO_2$: 274.0838; found: 274.0840.

2-(4-isopropylphenyl)isoindoline-1,3-dione (3d)[1,3]

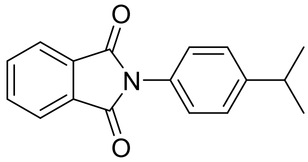

3d

Yield: 14.6 mg, (55%); white solid.

^{1}H NMR (400 MHz, CDCl$_3$) δ 7.98–7.93 (m, 2H), 7.82–7.76 (m, 2H), 7.39–7.32 (m, 4H), 2.97 (t, 1H), 1.29 (dd, *J* = 6.9, 1.5 Hz, 6H).

^{13}C NMR (101 MHz, CDCl$_3$) δ 167.6, 149.1, 134.5, 132.0, 129.3, 127.4, 126.6, 123.9, 34.1, 24.1.

HRMS (ESI): m/z [M + Na]$^+$ calcd for $C_{17}H_{15}NNaO_2$: 288.0995; found: 288.0997.

2-(2-Fluorophenyl)isoindoline-1,3-dione (3ea),

2-(3-fluorophenyl)isoindoline-1,3-dione (3eb),

2-(4-fluorophenyl)isoindoline-1,3-dione (3ec)[1,2]

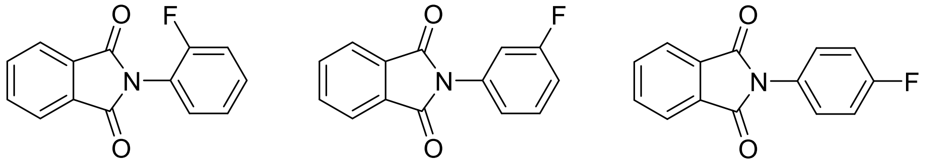

3ea **3eb** **3ec**

Yield: 13.2 mg, (54%); (**3ea**:**3eb**:**3ec** = 1:4:1); white solid. The isomer ratio was determined by ^{19}F NMR spectroscopy.

^{1}H NMR (400 MHz, CDCl$_3$): δ 8.00–7.94 (m, 2H), 7.84–7.79 (m, 2H), 7.50–7.35 (m, 1H [**3ea**],1H [**3eb**]), 7.32–7.18 (m, 3H), 7.14–7.09 (m, 1H [**3ec**]).

^{13}C NMR (101 MHz, CDCl$_3$): δ 164.8, 167.0, 166.7, 164.0, 163.4, 161.6, 160.9, 159.3, 156.8, 134.8, 134.7, 134.6, 131.7, 130.4, 130.3, 130.0, 128.6, 124.1 (2C), 122.2 (2C), 119.6, 119.5, 116.4, 116.2, 115.3, 115.1, 114.2, 114.0.

^{19}F NMR (471 MHz, CDCl$_3$): δ -111.17 [**3eb**], -113.06 [**3ec**], -118.68 [**3ea**].

HRMS (ESI): m/z [M + Na]$^+$ calcd for $C_{14}H_8FNNaO_2$: 264.0431; found: 264.0429.

2-(3-Chlorophenyl)isoindoline-1,3-dione (3fb),

2-(4-chlorophenyl)isoindoline-1,3-dione (3fc) [1,2]

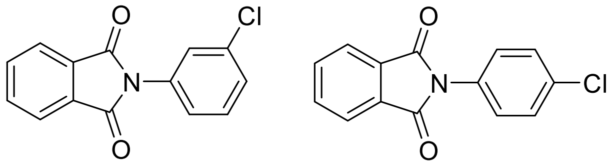

3fb **3fc**

Yield: 13.4 mg, (52%); (**3fb**:**3fc** = 2:1); white solid. The isomer ratio was determined by ^{1}H NMR spectroscopy.

^{1}H NMR (400 MHz, CDCl$_3$): δ 7.98–7.92 (m, 2H), 7.83–7.76 (m, 2H), 7.49 (dd, *J* = 4.0, 2.1 Hz, 1H [**3fb**]), 7.45 (d, *J* = 2.5 Hz, 2H [**3fc**]), 7.44–7.35 (m, 2H [**3fb**] + 2H [**3fc**]).

^{13}C NMR (101 MHz, CDCl$_3$): δ 167.1, 167.0, 134.8, 134.7, 134.0, 132.9, 131.8,

131.7, 130.3, 130.2, 129.5, 128.4, 127.8, 126.8, 124.7, 124.1, 124.0.

HRMS (ESI): m/z [M + Na]$^+$ calcd for $C_{14}H_8ClNNaO_2$: 280.0136 (100.0%), 282.0106 (32.0%); found: 280.0132 (100.0%), 282.0103 (32.0%).

2-(3-Bromophenyl)isoindoline-1,3-dione (3gb),

2-(4-bromophenyl)isoindoline-1,3-dione (3gc)[1,2]

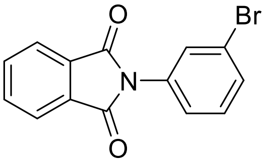

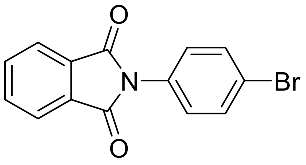

3gb **3gc**

Yield: 16.9 mg, (56%); (**3gb**:**3gc** = 1:2); white solid. The isomer ratio was determined by ^{1}H NMR spectroscopy.

^{1}H NMR (400 MHz, CDCl$_3$): δ 7.99–7.93 (m, 2H), 7.84–7.78 (m, 2H), 7.66–7.61 (m, 2H [**3gb**]), 7.54 (dt, *J* = 7.6, 1.6 Hz, 2H [**3gc**]), 7.44–7.39 (m, 2H [**3gb**]), 7.39–7.33 (m, 2H [**3gc**]).

^{13}C NMR (101 MHz, CDCl$_3$): δ 167.1, 134.8, 134.7, 133.1, 132.4, 131.8, 131.7, 131.3, 130.4, 129.7, 128.1, 125.2, 124.1, 124.0, 122.6, 122.0.

HRMS (ESI): m/z [M + Na]$^+$ calcd for $C_{14}H_8BrNNaO_2$: 323.9631 (100.0%), 325.9610 (97.3%); found: 323.9628 (100.0%), 325.9608 (97.3%).

2-(3-(Trifluoromethyl)phenyl)isoindoline-1,3-dione (3h)[1,2]

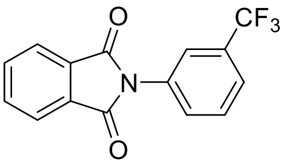

3h

Yield: 20.3 mg, (70%); white solid.

^{1}H NMR (400 MHz, CDCl$_3$): δ 8.01–7.94 (m, 2H), 7.84–7.79 (m, 2H), 7.79–7.60 (m, 4H).

^{13}C NMR (101 MHz, CDCl$_3$): δ 166.9, 134.9, 132.4, 131.6 (q, *J* = 33 Hz), 131.5,

129.8, 129.7, 126.6, 126.3, 124.8 (q, J = 4 Hz), 124.1, 123.6 (q, J = 273 Hz), 123.4 (q, J = 4 Hz).

^{19}F NMR (376 MHz, CDCl$_3$): δ -62.64.

HRMS (ESI): m/z [M + Na]$^+$ calcd for $C_{15}H_8F_3NNaO_2$: 314.0402; found: 314.0397.

2-(2-(Chloromethyl)phenyl)isoindoline-1,3-dione (3ia),

2-(3-(chloromethyl)phenyl)isoindoline-1,3-dione (3ib),

2-(4-(chloromethyl)phenyl)isoindoline-1,3-dione (3ic)[1,4]

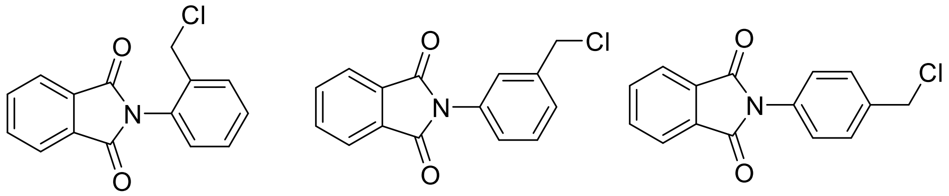

3ia 3ib 3ic

Yield: 10.8 mg, (40%); (**3ia**:**3ib**:**3ic** = 1:2:1); white solid. The isomer ratio was determined by ^{1}H NMR spectroscopy.

^{1}H NMR (400 MHz, CDCl$_3$) δ 8.01 – 7.92 (m, 2H), 7.91 – 7.73 (m, 2H), 7.61 – 7.27 (m, 4H), 4.64 (s, 2H [**3ib**]), 4.63 (s, 2H [**3ia**]), 4.54 (s, 2H [**3ic**]).

^{13}C NMR (101 MHz, CDCl$_3$) δ 168.0, 167.3, 138.7, 134.7, 134.5, 132.8, 132.0, 131.8, 130.9, 129.9, 129.6, 129.5, 128.3, 126.8, 126.7, 126.5, 124.1, 124.0, 123.7, 45.7, 43.0.

HRMS (ESI): m/z [M + Na]$^+$ calcd for $C_{15}H_{10}ClNNaO_2$: 294.0292 (100.0%), 296.0263 (32.0%); found 294.0292 (100.0%), 296.0263 (32.0%).

2-(3-(Bromomethyl)phenyl)isoindoline-1,3-dione (3jb),

2-(4-(bromomethyl)phenyl)isoindoline-1,3-dione (3jc) [5-7]

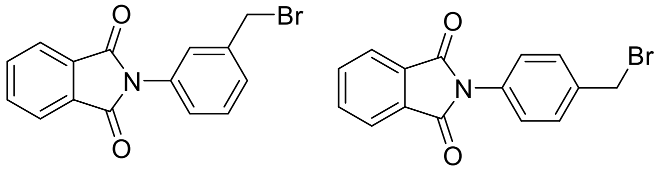

3jb 3jc

Yield: 13.2 mg, (42%); (**3jb**:**3jc** = 10:1); white solid. The isomer ratio was determined by ^{1}H NMR spectroscopy.

^{1}H NMR (400 MHz, $CDCl_3$): δ 7.99–7.93 (m, 2H), 7.84–7.78 (m, 2H), 7.56–7.36 (m, 2H), 4.54 (s, 2H [**3jb**]), 4.43 (s, 2H [**3jc**]).

^{13}C NMR (101 MHz, $CDCl_3$): δ 167.2, 139.0, 134.7, 134.6, 132.2, 131.8, 130.0 (2C), 129.7, 129.5, 128.8, 128.7, 127.1, 126.5, 124.1, 124.0, 123.6, 32.7, 29.6.

HRMS (ESI): m/z $[M + Na]^+$ calcd for $C_{15}H_{10}BrNNaO_2$: 337.9787 (100.0%), 339.9767 (97.3%); found: 337.9788 (100.0%), 339.9768 (97.3%).

2-(3,4-Dichlorophenyl)isoindoline-1,3-dione (3k)[1]

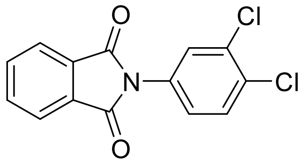

3k

Yield: 8.7 mg, (30%); white solid.

^{1}H NMR (400 MHz, $CDCl_3$): δ 7.99–7.93 (m, 2H), 7.84–7.79 (m, 2H), 7.63 (dd, *J* = 2.4, 0.9 Hz, 1H), 7.57 (dd, *J* = 8.6, 0.9 Hz, 1H), 7.36 (ddd, *J* = 8.6, 2.4, 0.9 Hz, 1H).

^{13}C NMR (101 MHz, $CDCl_3$): δ 166.8, 134.9, 133.2, 132.3, 131.6, 131.3, 130.8, 129.0, 128.3, 125.7, 124.1, 117.7.

HRMS (ESI): m/z $[M + Na]^+$ calcd for $C_{14}H_7Cl_2NaO_2$: 313.9752 (100.0%), 315.9722(63.9%); found: 313.9791 (100.0%), 315.9896 (63.9%).

2-(5-Chloro-2-methylphenyl)isoindoline-1,3-dione (3la)，

2-(2-chloro-5-methylphenyl)isoindoline-1,3-dione (3lb)[1,8]

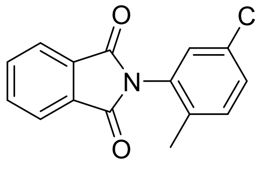

3la

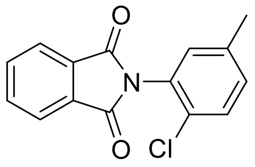

3lb

Yield: 14.9 mg, (56%); (**3la**:**3lb** = 3:1); white solid. The isomer ratio was determined by ^{1}H NMR spectroscopy.

^{1}H NMR (400 MHz, $CDCl_3$): δ 8.00–7.92 (m, 2H), 7.85–7.77 (m, 2H), 7.44 (d, *J* =

8.2 Hz, 1H [**3lb**]), 7.35 (dd, *J* = 8.3, 2.1 Hz, 1H [**3la**]), 7.30 (d, *J* = 8.3 Hz, 1H [**3la**]), 7.24 (d, *J* = 2.1 Hz, 1H [**3lb**]), 7.23 (d, *J* = 2.2 Hz, 1H [**3la**]), 7.16 (d, *J* = 2.1 Hz, 1H [**3lb**]), 2.39 (s, 3H [**3lb**]), 2.17 (s, 3H [**3la**]).

^{13}C NMR (101 MHz, CDCl$_3$): δ 167.0, 166.9, 138.2, 135.3, 134.7, 134.6, 132.2, 132.1, 132.0 (2C), 131.7, 131.6, 131.2, 130.1, 129.7, 129.0, 124.0, 20.9, 17.8.

HRMS (ESI): m/z [M + Na]$^+$ calcd for $C_{15}H_{10}ClNNaO_2$: 294.0292 (100.0%), 296.0263 (32.0%); found: 294.0290 (100.0%), 296.0264 (32.0%).

2-(2,5-Dimethylphenyl)isoindoline-1,3-dione (3m)[1,2]

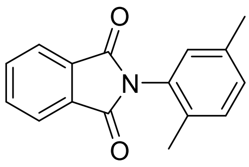

3m

Yield: 20.1 mg, (80%); white solid.

^{1}H NMR (600 MHz, CDCl$_3$): δ 7.97–7.94 (m, 2H), 7.81–7.77 (m, 2H), 7.25 (d, *J* = 7.6 Hz, 1H), 7.19 (dd, *J* = 8.0, 1.8 Hz, 1H), 7.03 – 7.02 (m, 1H), 2.36 (s, 3H), 2.16 (s, 3H).

^{13}C NMR (101 MHz, CDCl$_3$): δ 167.5, 136.7, 134.4, 133.3, 132.1, 131.0, 130.4 (2C), 129.2, 123.8, 20.9, 17.6.

HRMS (ESI): m/z [M + Na]$^+$ calcd for $C_{16}H_{13}NNaO_2$: 274.0838; found: 274.0836.

2-(2,3-Dimethylphenyl)isoindoline-1,3-dione (3na)，

2-(3,4-dimethylphenyl)isoindoline-1,3-dione (3nb) [1,2]

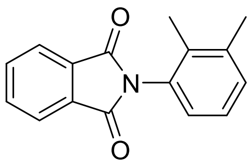

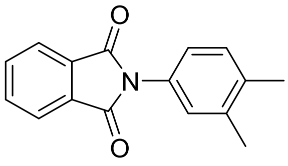

3na **3nb**

Yield: 15.5 mg, (62%); (**3na**:**3nb** = 1:1); white solid. The isomer ratio was determined by ^{1}H NMR spectroscopy.

^{1}H NMR (400 MHz, CDCl$_3$): δ 7.96–7.89 (m, 2H), 7.80–7.73 (m, 2H), 7.27–7.17 (m, 2H [**3na**] + 1H [**3nb**]), 7.16 (d, *J* = 2.2 Hz, 1H [**3nb**]), 7.13–7.09 (m, 1H [**3nb**]), 7.03 (d, *J* = 1.6 Hz, 1H [**3na**]), 2.33 (s, 3H [**3na**]), 2.29 (d, *J* = 2.9 Hz, 6H [**3nb**]), 2.06 (s, 3H [**3na**]).

^{13}C NMR (101 MHz, CDCl$_3$): δ 167.7 (2C), 138.5, 137.8, 137.2, 135.2, 134.4 (2C), 132.2, 132.0, 131.1, 130.6, 130.4, 129.2, 127.9, 126.4 (2C), 124.3, 123.9, 123.8, 20.6, 20.0, 19.7, 14.8.

HRMS (ESI): m/z [M + Na]$^+$ calcd for $C_{16}H_{13}NNaO_2$: 274.0838; found: 274.0832.

2-(2,6-Dimethylphenyl)isoindoline-1,3-dione (3oa),

2-(2,4-dimethylphenyl)isoindoline-1,3-dione (3ob)[1,2]

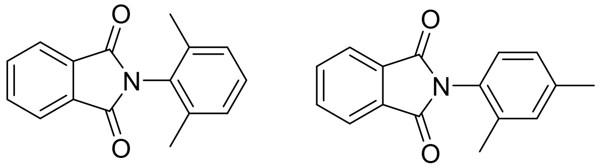

3oa **3ob**

Yield: 16.3 mg, (65%); (**3oa**:**3ob** = 1:8); white solid. The isomer ratio was determined by ^{1}H NMR spectroscopy.

^{1}H NMR (400 MHz, CDCl$_3$): δ 7.98–7.93 (m, 2H), 7.83–7.76 (m, 2H), 7.30–7.28 (m, 1H [**3oa**]), 7.21–7.17 (m, 2H [**3oa**] + 1H [**3ob**]), 7.14 (dd, *J* = 7.9 Hz, 1H [**3ob**]), 7.09 (d, *J* = 8.0 Hz, 1H [**3ob**]), 2.38 (s, 3H [**3ob**]), 2.17 (d, *J* = 1.4 Hz, 6H [**3oa**], 3H [**3ob**]).

^{13}C NMR (101 MHz, CDCl$_3$): δ 167.7, 139.6, 137.0, 136.3, 134.5, 134.4, 132.2, 132.0, 129.6, 128.6, 128.0, 127.8, 123.9, 123.8, 21.3, 18.2, 18.0.

HRMS (ESI): m/z [M + Na]$^+$ calcd for $C_{16}H_{13}NNaO_2$: 274.0838; found: 274.0840.

2-Mesitylisoindoline-1,3-dione (3p) [1,2]

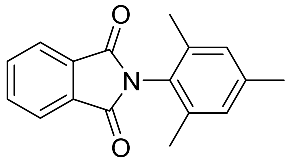

3p

Yield: 15.4 mg, (58%); white solid.

^{1}H NMR (400 MHz, CDCl$_3$): δ 7.99–7.94 (m, 2H), 7.83–7.77 (m, 2H), 7.01 (s, 2H), 2.33 (s, 3H), 2.12 (s, 6H).

^{13}C NMR (101 MHz, CDCl$_3$): δ 167.6, 139.5, 136.6, 134.4, 132.2, 129.4, 127.2, 123.9, 21.3, 18.1.

HRMS (ESI): m/z [M + Na]$^+$ calcd for $C_{17}H_{15}NNaO_2$: 288.0995; found: 288.0995.

2-(Furan-2-yl)isoindoline-1,3-dione (3q)[1]

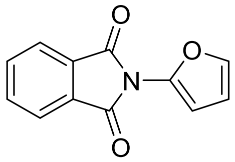

3q

Yield: 7.1 mg, (33%); light yellow solid.

^{1}H NMR (600 MHz, CDCl$_3$): δ 8.00–7.95 (m, 2H), 7.85–7.80 (m, 2H), 7.47 (dd, J = 2.1, 1.0 Hz, 1H), 6.55 (dd, J = 3.3, 2.1 Hz, 1H), 6.46 (dd, J = 3.4, 1.0 Hz, 1H).

^{13}C NMR (101 MHz, CDCl$_3$): δ 166.3, 141.8, 138.0, 134.9, 131.8, 131.3, 129.0, 124.3, 111.6, 106.8.

HRMS (ESI): m/z [M + Na]$^+$ calcd for $C_{12}H_7NNaO_3$: 236.0318; found: 236.0316.

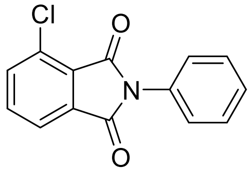

3r

4-Chloro-2-phenylisoindoline-1,3-dione (3r)[1]

Yield: 20.8 mg, (81%); white solid.

^{1}H NMR(400 MHz, $CDCl_3$): δ 7.91–7.84 (m, 1H), 7.75–7.68 (m, 2H), 7.51 (dd, *J* = 8.5, 6.9 Hz, 2H), 7.43 (dd, *J* = 8.1, 2.4 Hz, 3H).

^{13}C NMR (101 MHz, $CDCl_3$): δ 166.0, 165.0, 136.3, 135.4, 134.0, 132.0, 131.4, 129.3, 128.4, 127.5, 126.7, 122.4.

HRMS (ESI): m/z [M + Na]$^+$ calcd for $C_{14}H_8ClNNaO_2$: 280.0136 (100.0%), 282.0106 (32.0%); found: 280.0132 (100.0%), 282.0103 (32.0%).

5-Chloro-2-phenylisoindoline-1,3-dione (3s) [1,9]

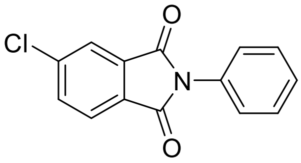

3s

Yield: 19.2 mg, (75%); white solid.

^{1}H NMR(400 MHz, $CDCl_3$): δ 7.94–7.87 (m, 2H), 7.75 (dd, *J* = 8.0, 1.8 Hz, 1H), 7.55–7.48 (m, 2H), 7.45–7.39 (m, 3H).

^{13}C NMR (101 MHz, $CDCl_3$): δ 166.4, 166.1, 141.3, 134.6, 133.5, 131.5, 129.9, 129.3, 128.5, 126.6, 125.2, 124.3.

HRMS (ESI): m/z [M + Na]$^+$ calcd for $C_{14}H_8ClNNaO_2$: 280.0136 (100.0%), 282.0106 (32.0%); found: 280.0133 (100.0%), 282.0105 (32.0%).

5-Bromo-2-phenylisoindoline-1,3-dione (3t)[1,10]

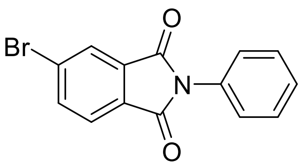

3t

Yield: 25.1 mg, (83%); white solid.

^{1}H NMR(400 MHz, $CDCl_3$): δ 8.10 (d, *J* = 1.7 Hz, 1H), 7.93 (dd, *J* = 7.9, 1.7 Hz, 1H), 7.82 (d, *J* = 7.9 Hz, 1H), 7.52 (t, *J* = 7.7 Hz, 2H), 7.46–7.39 (m, 3H). 7.40 (d, *J* =

7.8 Hz, 3H).

^{13}C NMR (101 MHz, CDCl$_3$): δ 166.6, 137.6, 133.5, 131.5, 130.4, 129.5, 129.3, 128.5, 127.3, 126.6, 125.3.

HRMS (ESI): m/z [M + Na]$^+$ calcd for $C_{14}H_8BrNNaO_2$: 323.9631 (100.0%), 325.9610 (97.3%); found: 323.9629 (100.0%), 325.9608 (97.3%).

5-Methoxy-2-phenylisoindoline-1,3-dione (3u) [11]

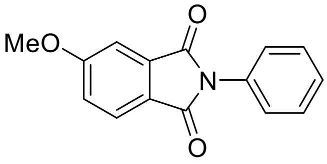

3u

Yield: 9.9 mg, (39%); white solid.

^{1}H NMR(400 MHz, CDCl$_3$): δ 7.85 (dt, *J* = 8.3, 2.6 Hz, 1H), 7.55–7.46 (m, 2H), 7.46–7.35 (m, 4H), 7.27–7.20 (m, 1H), 3.99–3.92 (m, 3H).

^{13}C NMR (101 MHz, CDCl$_3$): δ 165.1, 134.6, 132.0, 129.2, 128.1, 126.7, 125.6, 123.8, 120.6, 108.3, 56.3.

HRMS (ESI): m/z [M + Na]$^+$ calcd for $C_{15}H_{11}NNaO_3$: 276.0631; found: 276.0625.

4. References

[1] Lv, B.; Gao, P.; Zhang, S.; Jia, X.; Wang, M.; Yuan, Y. *Org. Chem. Front.* **2021,** *8*, 5440-5445. doi:10.1039/D1QO00710F

[2] Kuribara, T.; Nakajima, M.; Nemoto, T. *Org. Lett.* **2020,** *22*, 2235-2239. doi:10.1021/acs.orglett.0c00433

[3] Shrestha, R.; Mukherjee, P.; Tan, Y.; Litman, Z. C.; Hartwig, J. F. *J. Am. Chem. Soc.* **2013,** *135,* 8480-8483. doi:10.1021/ja4032677

[4] Marchetti, L.; Kantak, A.; Davis, R.; DeBoef, B. *Org. Lett.* **2015,** *17*, 358-361. doi:10.1021/ol5034805

[5] Pingali, S. R. K.; Upadhyay, S. K.; Jursic, B. S. *Green Chem.* **2011,** *13*, 928-933. doi:10.1039/C0GC00794C

[6] Upadhyay, S. K.; Jursic, B. S. *Synth. Commun.* **2011,** *41,* 3177-3185. doi:10.1080/00397911.2010.517378

[7] Zhang, X.; Huang, D.; Chen, Y.-S.; Holm, R. H. *Inorg. Chem.* **2012,** *51,* 11017-11029. doi:10.1021/ic301506x

[8] Guillaumel, J.; Léonce, S.; Pierré, A.; Renard, P.; Pfeiffer, B.; Arimondo, P. B.; Monneret, C. *Eur. J. Med. Chem.* **2006,** *41*, 379-386. doi: 10.1016/j.ejmech.2005.10.008

[9] De Sarkar, S.; Ackermann, L. *Chem. Eur. J.* **2014,** *20,* 13932-13936. doi: 10.1002/chem.201404261

[10] Wolff, O.; Waldvogel, S. R. *Synthesis* **2007,** *2007,* 761-765. doi:10.1055/s-2007-965919

[11] Cabrero-Antonino, J. R.; Adam, R.; Papa, V.; Holsten, M.; Junge, K.; Beller, M. *Chem. Sci.* **2017,** *8,* 5536-5546. doi:10.1039/C7SC01175J

5. Copies of ^{1}HNMR, ^{13}CNMR and ^{19}F NMR spectra

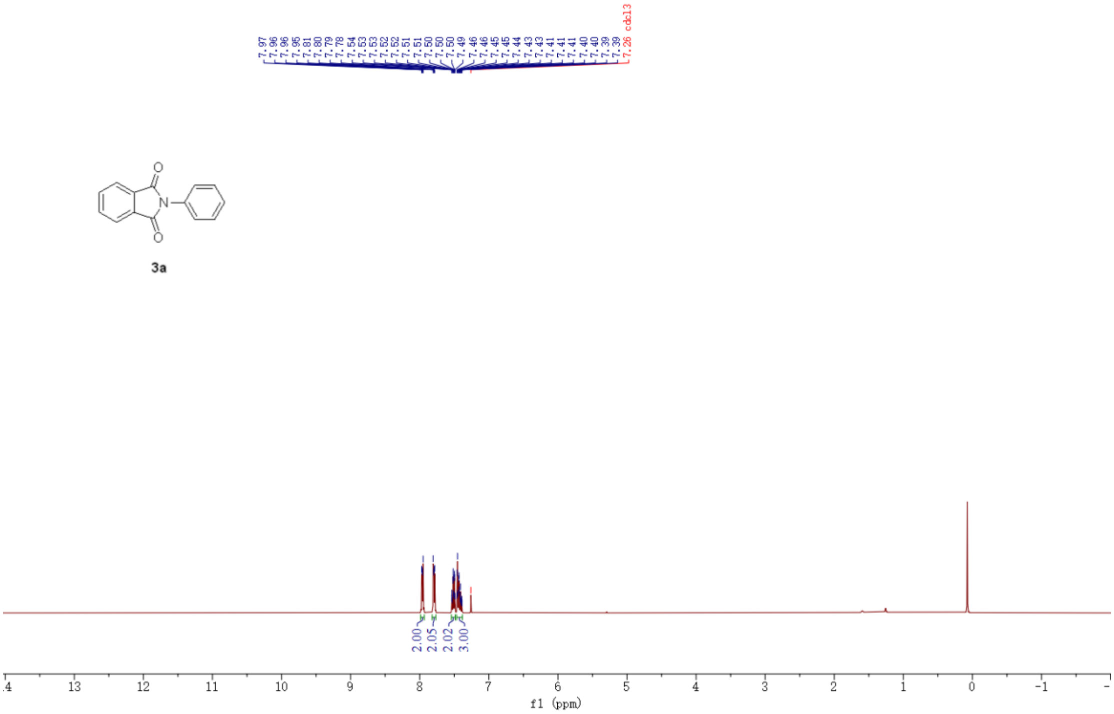

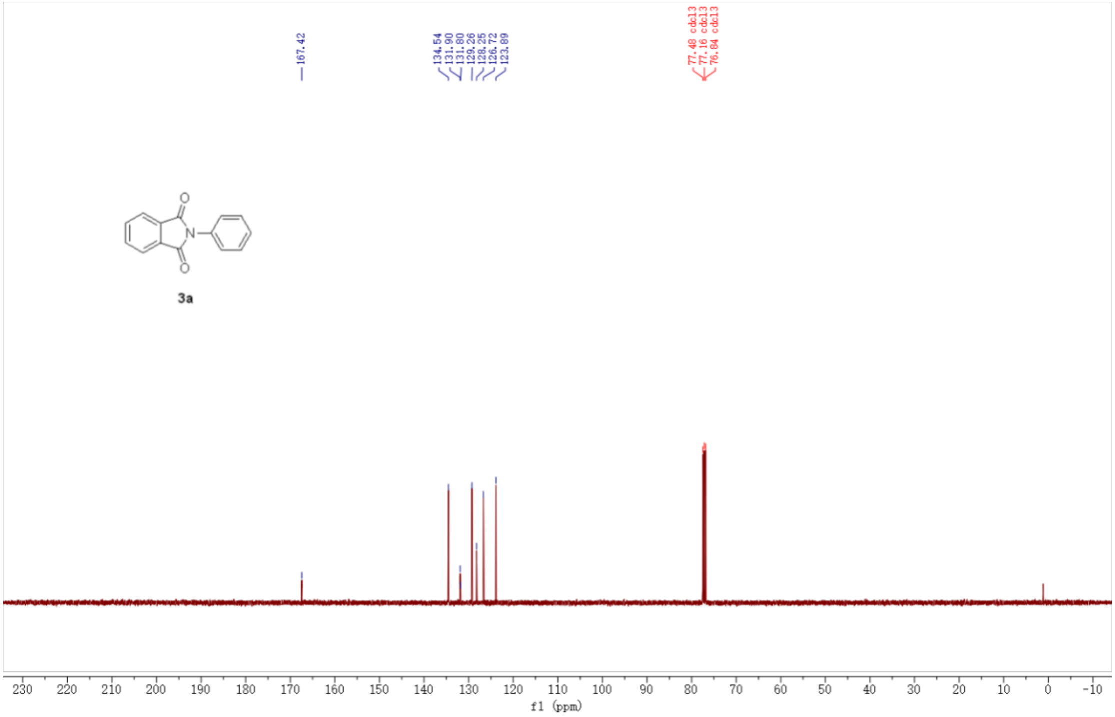

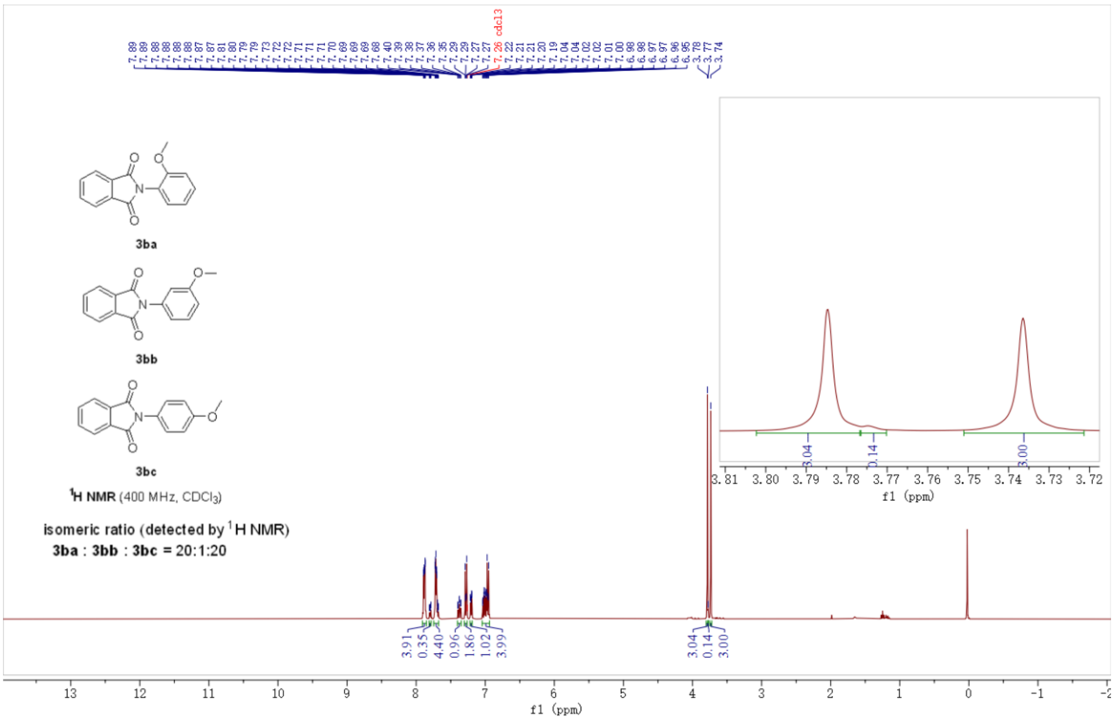
3ba
3bb
3bc
1H NMR (400 MHz, CDCl3)
isomeric ratio (detected by 1H NMR)
3ba : 3bb : 3bc = 20:1:20
f1 (ppm)

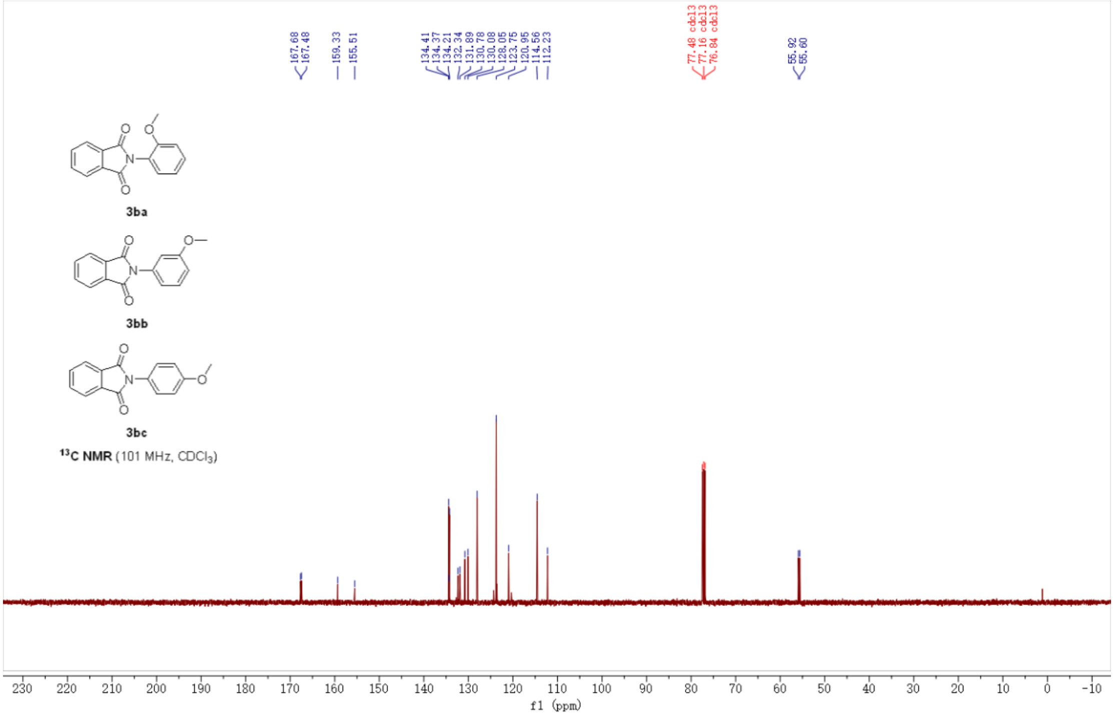
167.68
167.48
159.33
155.51
134.41
134.37
134.21
132.34
131.89
130.78
130.08
128.05
123.75
120.95
114.56
112.23
77.48 cdcl3
77.16 cdcl3
76.84 cdcl3
55.92
55.60
3ba
3bb
3bc
13C NMR (101 MHz, CDCl3)
f1 (ppm)

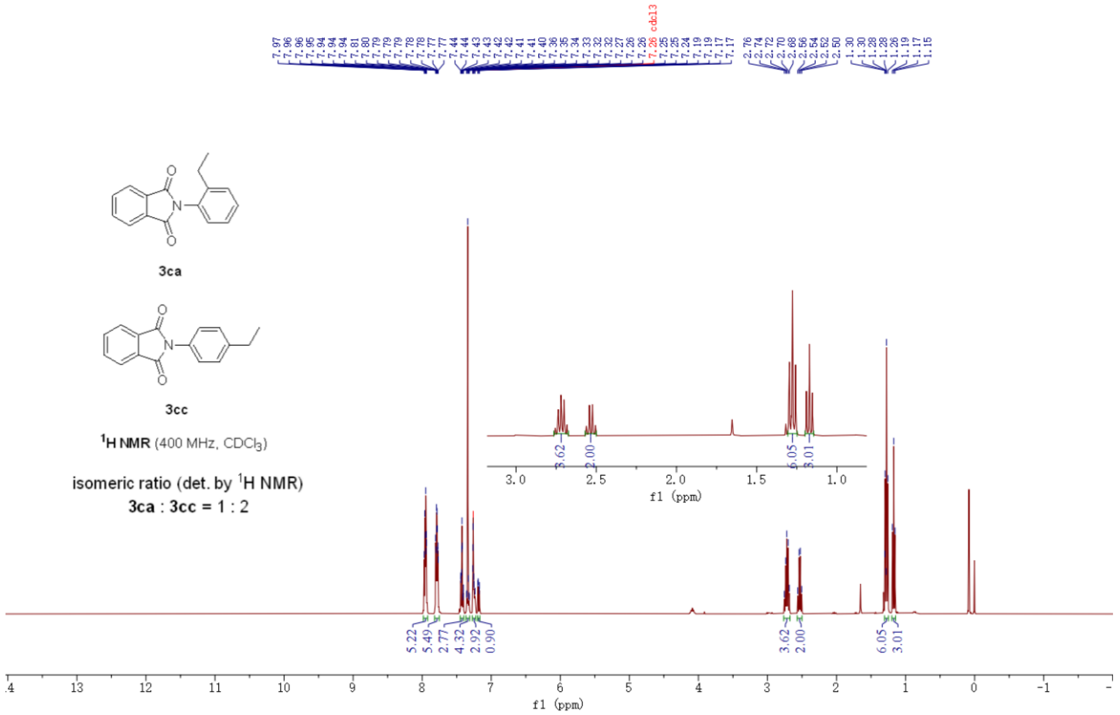
3ca
3cc
¹H NMR (400 MHz, CDCl₃)
isomeric ratio (det. by ¹H NMR)
3ca : 3cc = 1 : 2
f1 (ppm)

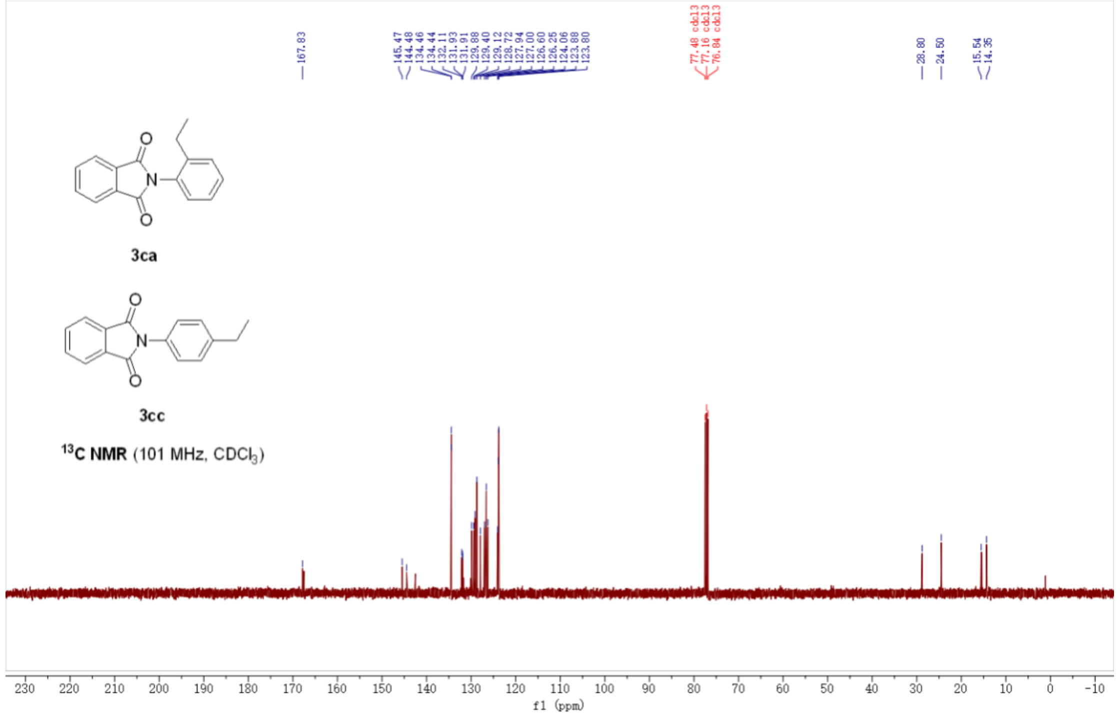
167.83
145.47
144.48
134.46
134.44
132.11
131.93
131.91
129.88
129.40
129.12
128.72
127.94
127.00
126.60
126.25
124.06
123.88
123.80
77.48 cdcl3
77.16 cdcl3
76.84 cdcl3
28.80
24.50
15.54
14.35
3ca
3cc
¹³C NMR (101 MHz, CDCl₃)
f1 (ppm)

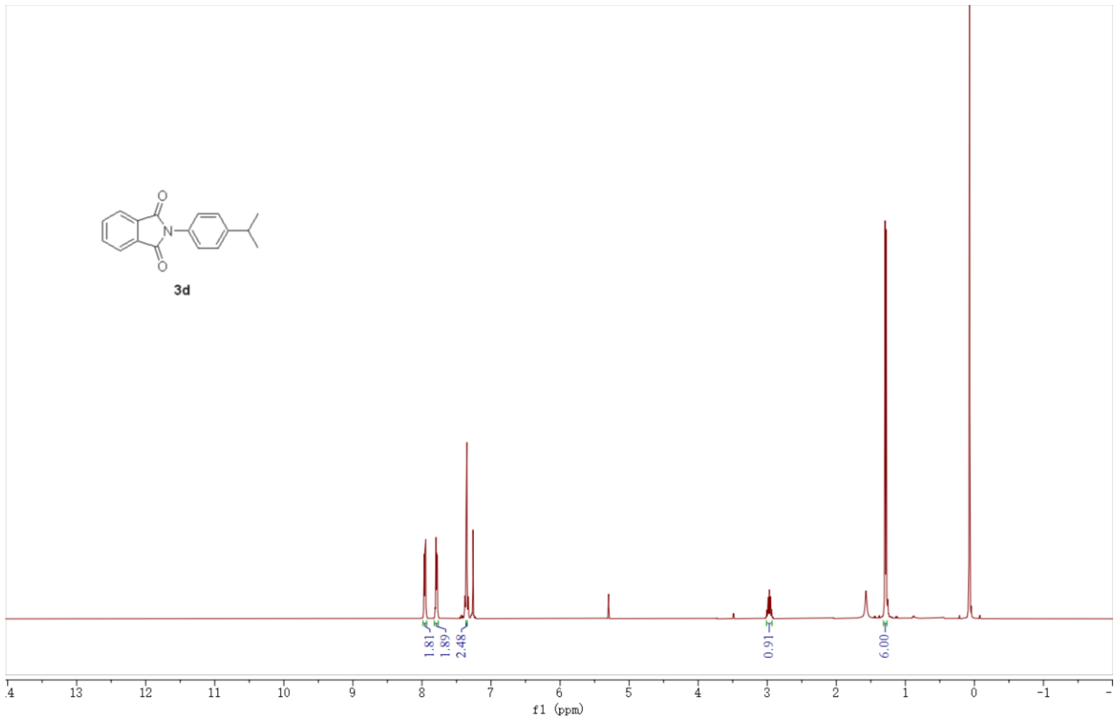
3d
1.81
1.89
2.48
0.91
6.00
f1 (ppm)

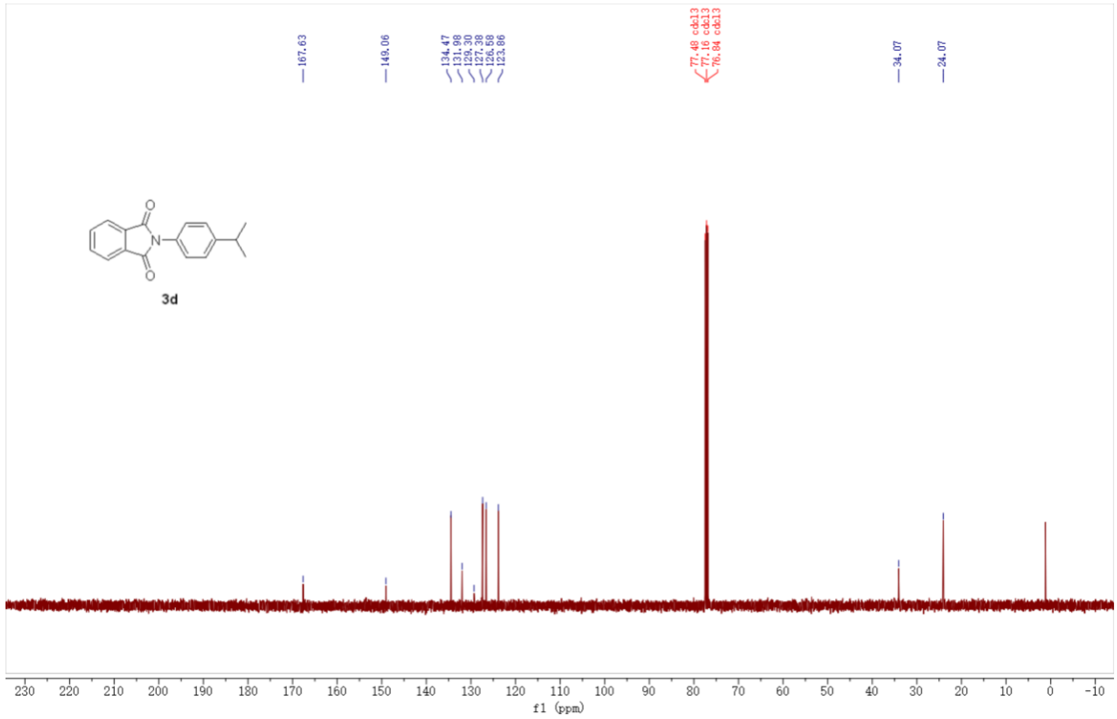
167.63
149.06
134.47
131.98
129.30
127.38
126.58
123.86
77.48 cdcl3
77.16 cdcl3
76.84 cdcl3
34.07
24.07
3d
f1 (ppm)

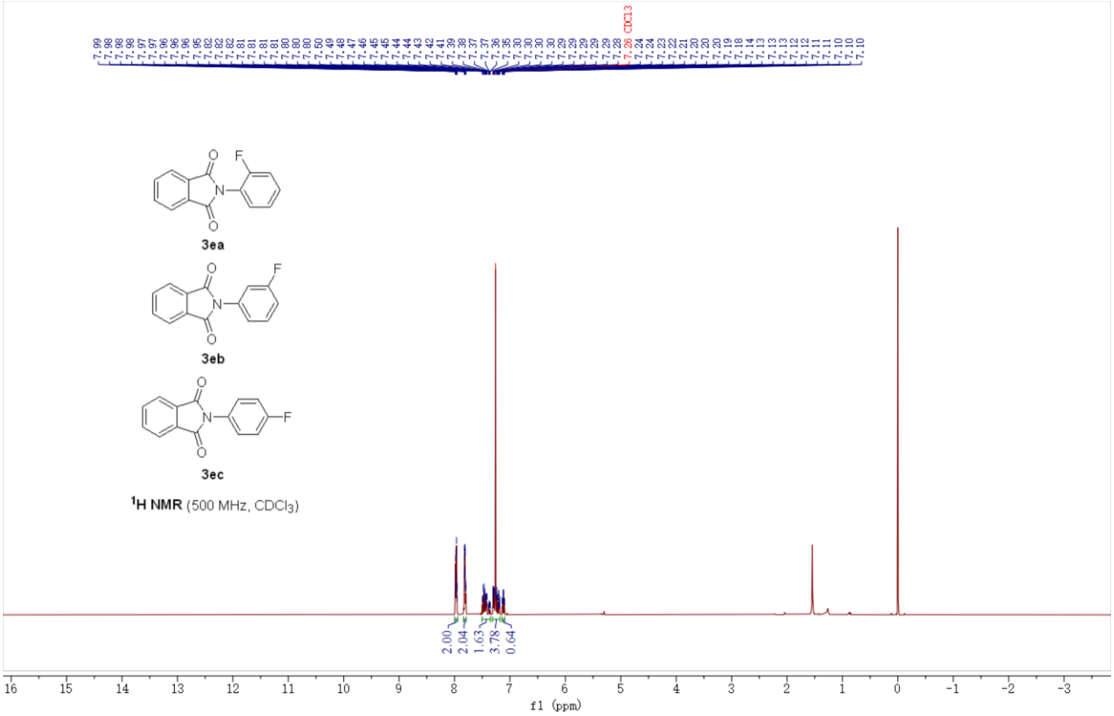
3ea
3eb
3ec
1H NMR (500 MHz, CDCl3)
f1 (ppm)

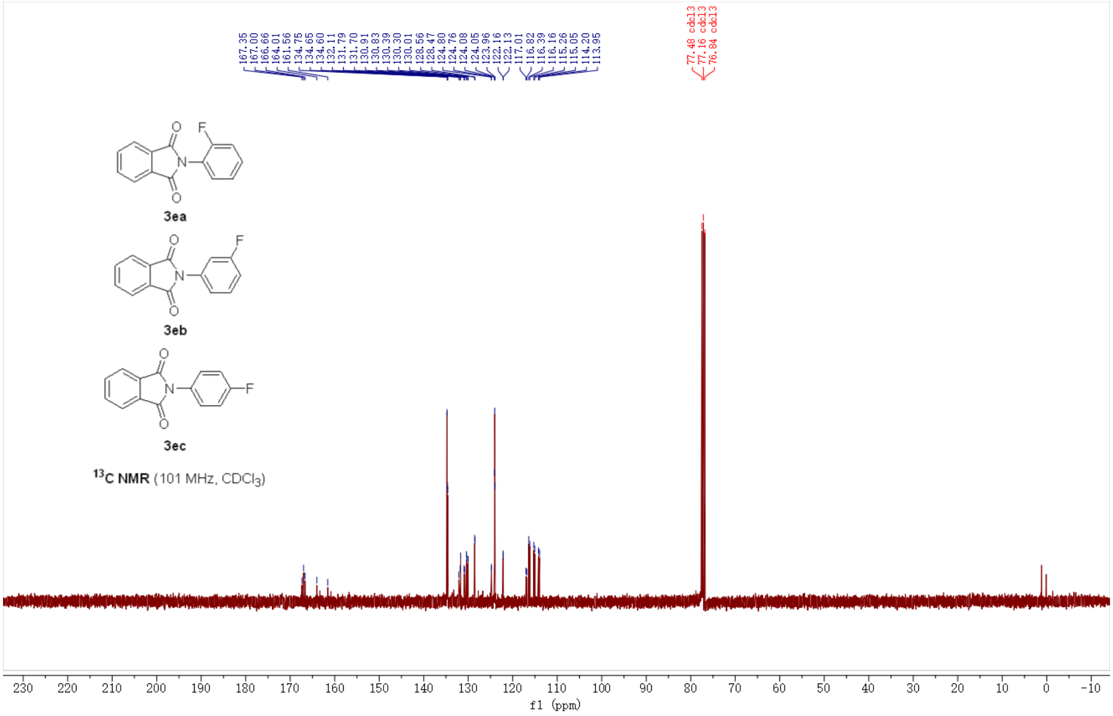
3ea
3eb
3ec
13C NMR (101 MHz, CDCl3)
f1 (ppm)

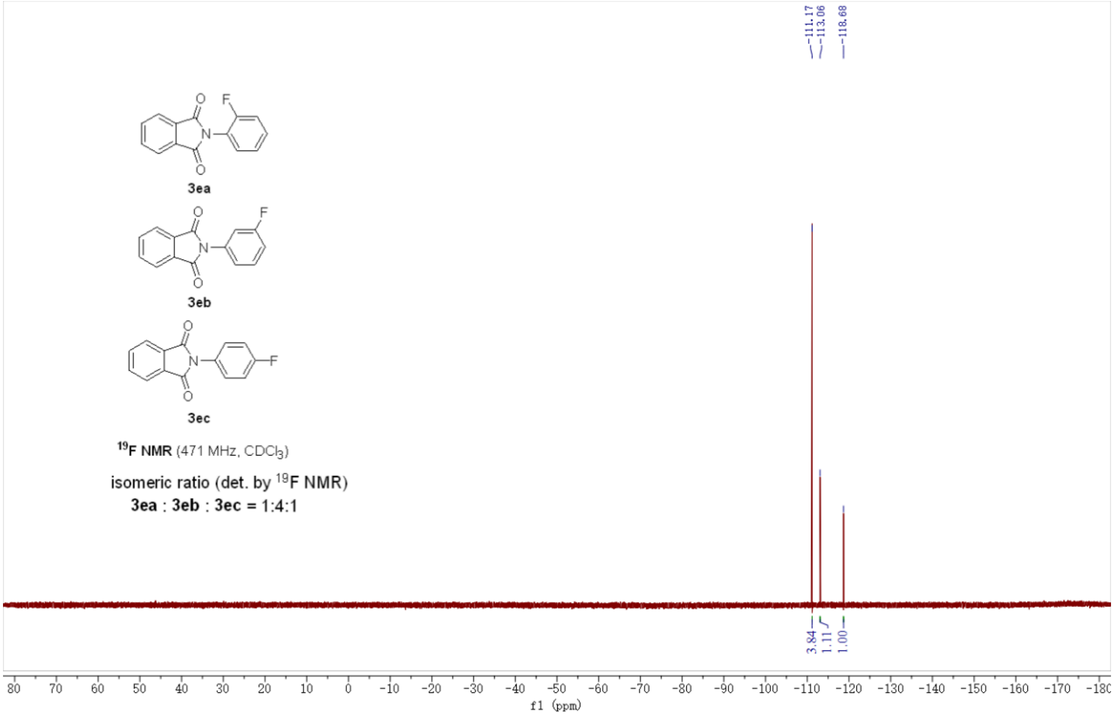
3ea
3eb
3ec
19F NMR (471 MHz, CDCl3)
isomeric ratio (det. by 19F NMR)
3ea : 3eb : 3ec = 1:4:1
-111.17
-113.06
-118.68
3.84
1.11
1.00
f1 (ppm)

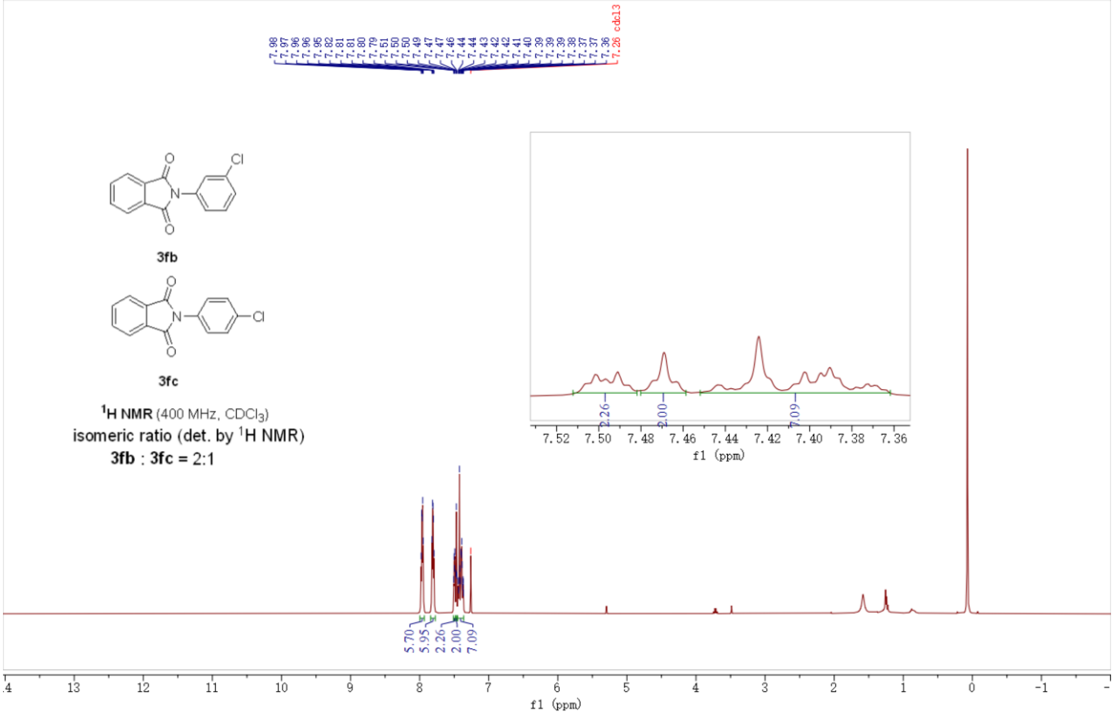
3fb
3fc
1H NMR (400 MHz, CDCl3)
isomeric ratio (det. by 1H NMR)
3fb : 3fc = 2:1
7.26 cdcl3
5.70
5.95
2.26
2.00
7.09
f1 (ppm)

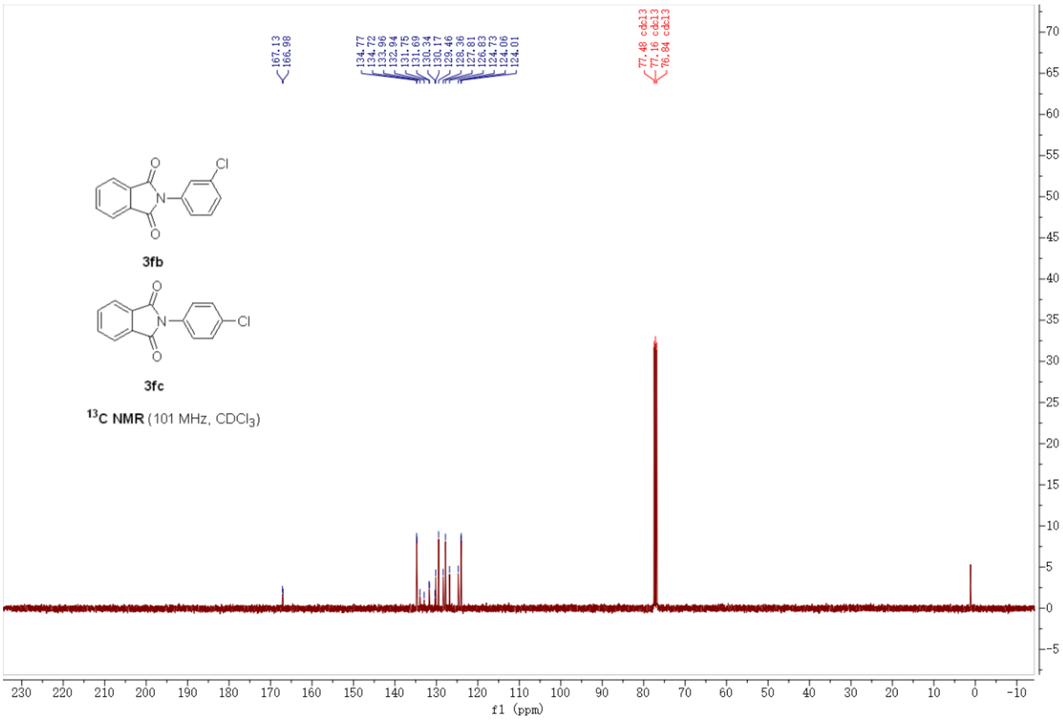
167.13
166.98
134.77
134.72
133.96
132.94
131.75
131.69
130.34
130.17
128.46
128.36
127.81
126.83
124.73
124.06
124.01
77.48 cdcl3
77.16 cdcl3
76.84 cdcl3
3fb
3fc
^{13}C NMR (101 MHz, CDCl$_3$)
f1 (ppm)

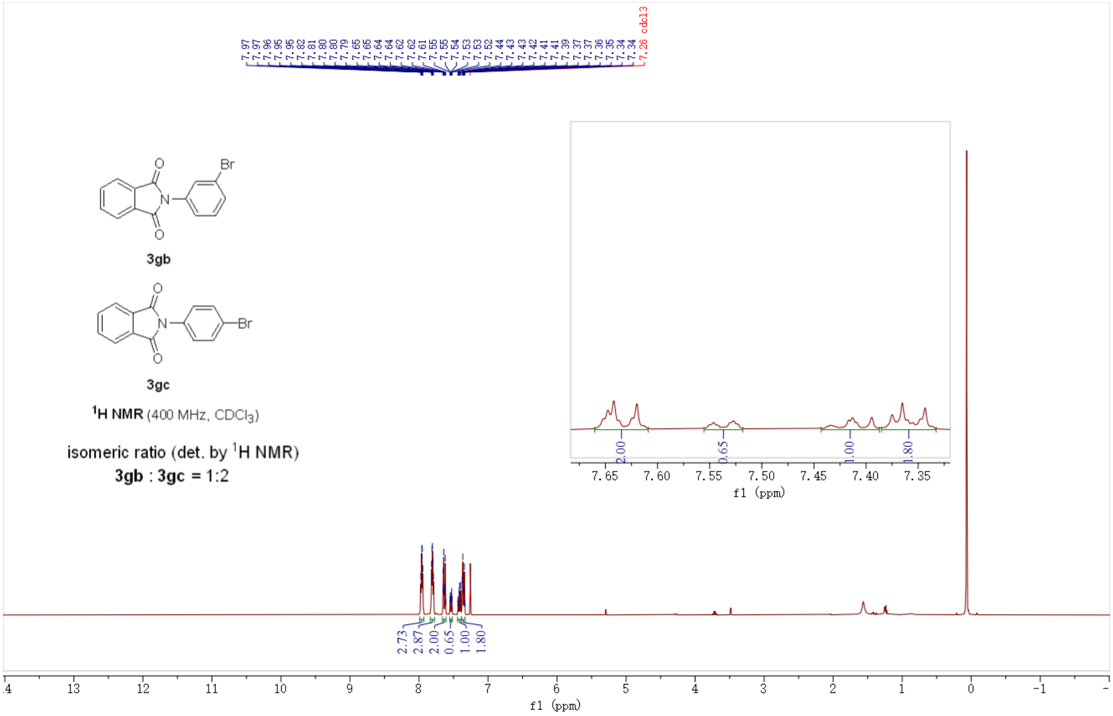
7.26 cdcl3
3gb
3gc
^{1}H NMR (400 MHz, CDCl$_3$)
isomeric ratio (det. by ^{1}H NMR)
3gb : 3gc = 1:2
2.73
2.87
2.00
0.65
1.00
1.80
f1 (ppm)

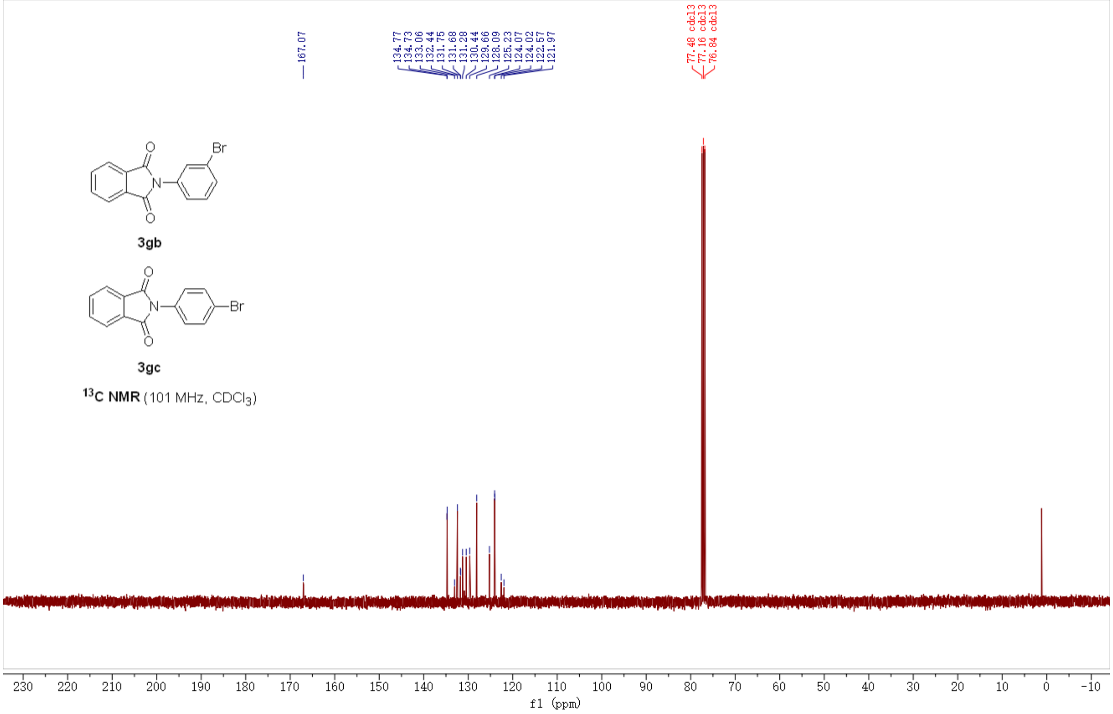
167.07
134.77
134.73
133.06
132.44
131.75
131.68
131.28
130.44
129.66
128.09
125.23
124.07
124.02
122.57
121.97
77.48 cdcl3
77.16 cdcl3
76.84 cdcl3
Br
N
O
O
3gb
Br
N
O
O
3gc
13C NMR (101 MHz, CDCl3)
f1 (ppm)

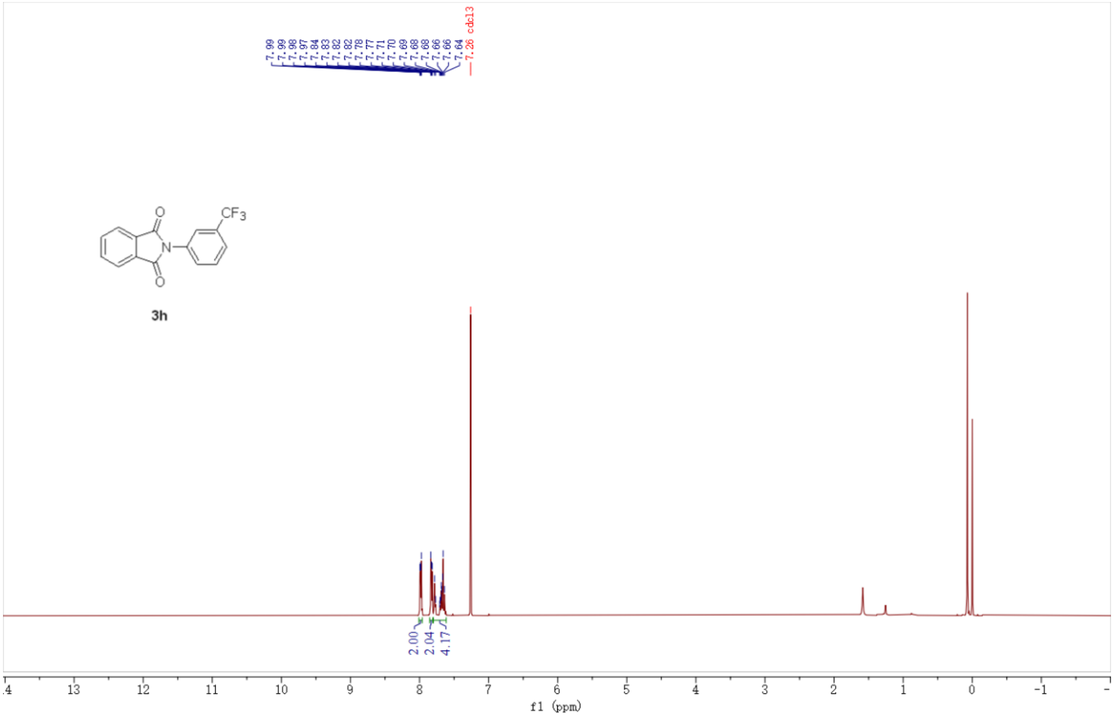
7.99
7.99
7.98
7.97
7.84
7.83
7.82
7.82
7.78
7.77
7.71
7.70
7.69
7.68
7.68
7.66
7.66
7.64
7.26 cdcl3
CF3
N
O
O
3h
2.00
2.04
4.17
f1 (ppm)

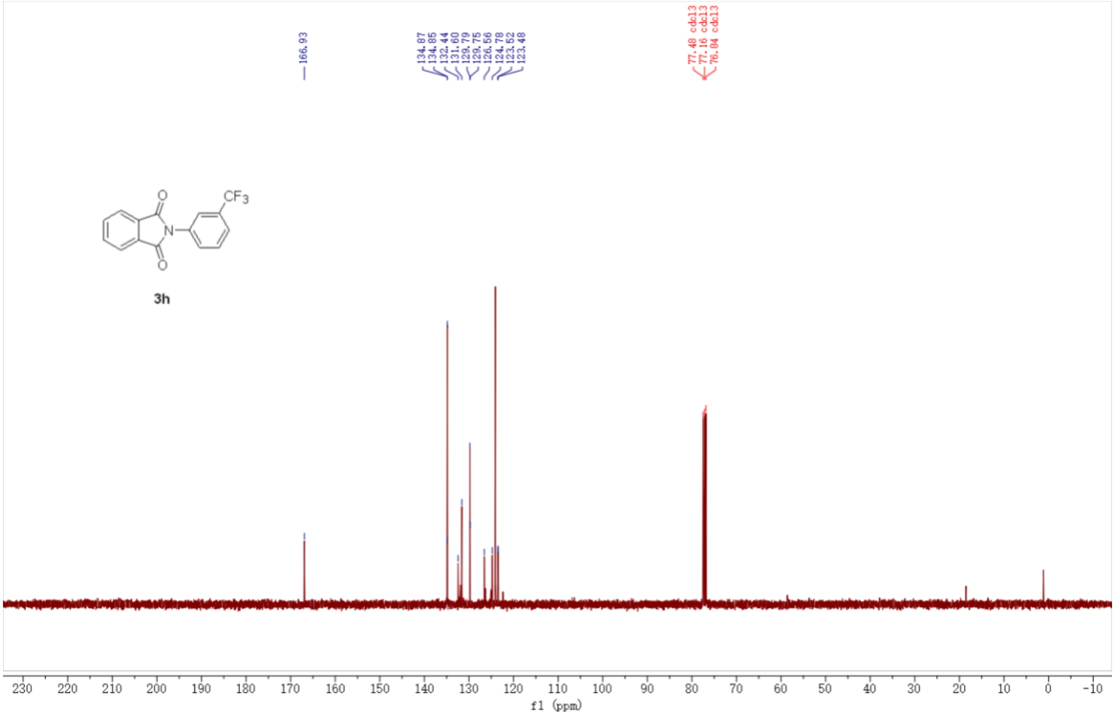

166.93
134.87
134.85
132.44
131.60
129.79
129.75
126.56
124.78
123.52
123.48
77.48 cdcl3
77.16 cdcl3
76.84 cdcl3
CF3
O
N
O
3h
f1 (ppm)

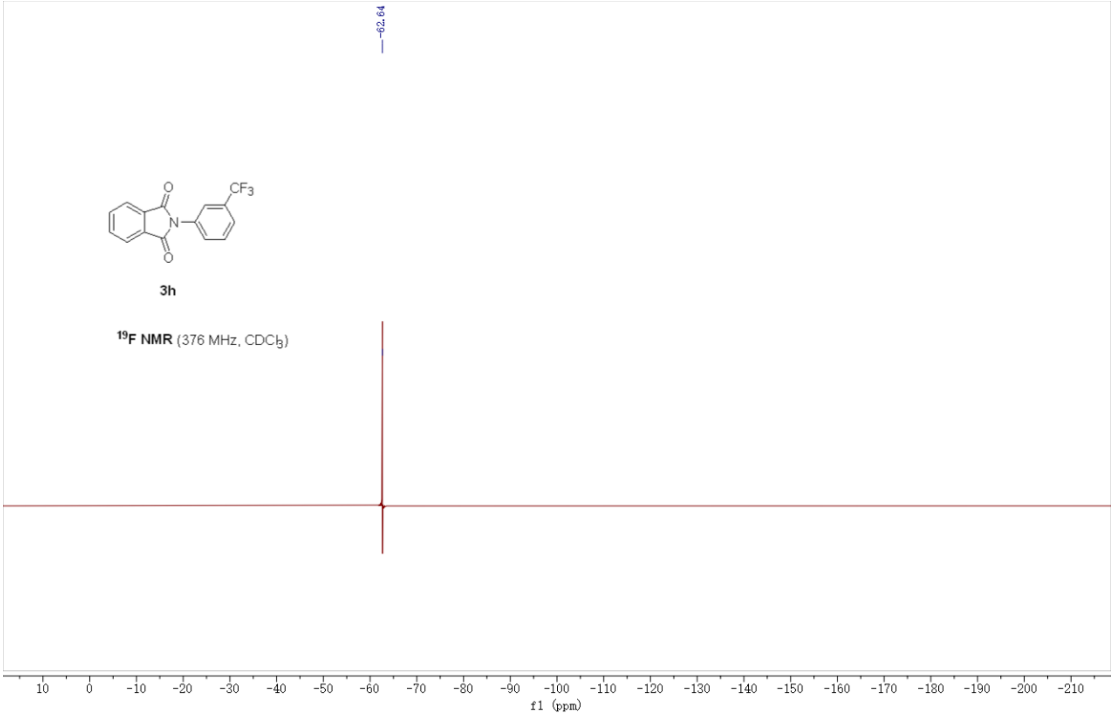

-62.64
CF3
O
N
O
3h
19F NMR (376 MHz, CDCl3)
f1 (ppm)

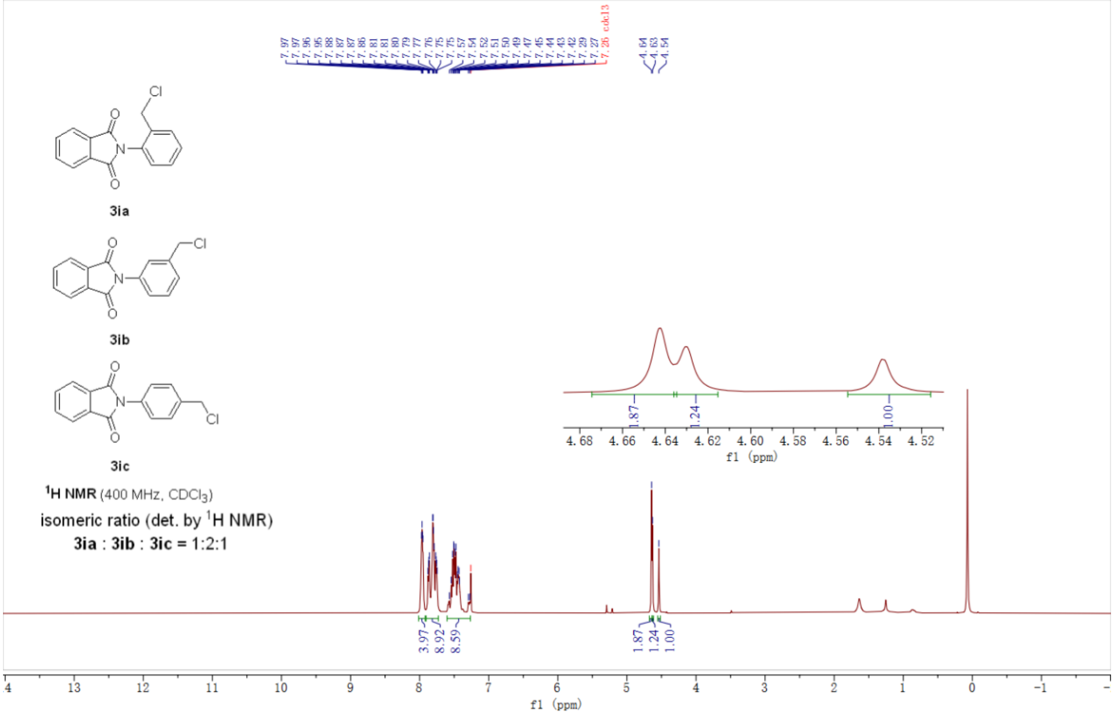
3ia
3ib
3ic
¹H NMR (400 MHz, CDCl₃)
isomeric ratio (det. by ¹H NMR)
3ia : 3ib : 3ic = 1:2:1
7.97 7.97 7.96 7.95 7.88 7.87 7.87 7.86 7.81 7.81 7.80 7.79 7.77 7.76 7.75 7.75 7.57 7.54 7.52 7.51 7.50 7.49 7.47 7.45 7.44 7.43 7.42 7.29 7.27 7.26 cdcl3
4.64 4.63 4.54
1.87 1.24 1.00
3.97 8.92 8.59 1.87 1.24 1.00
f1 (ppm)

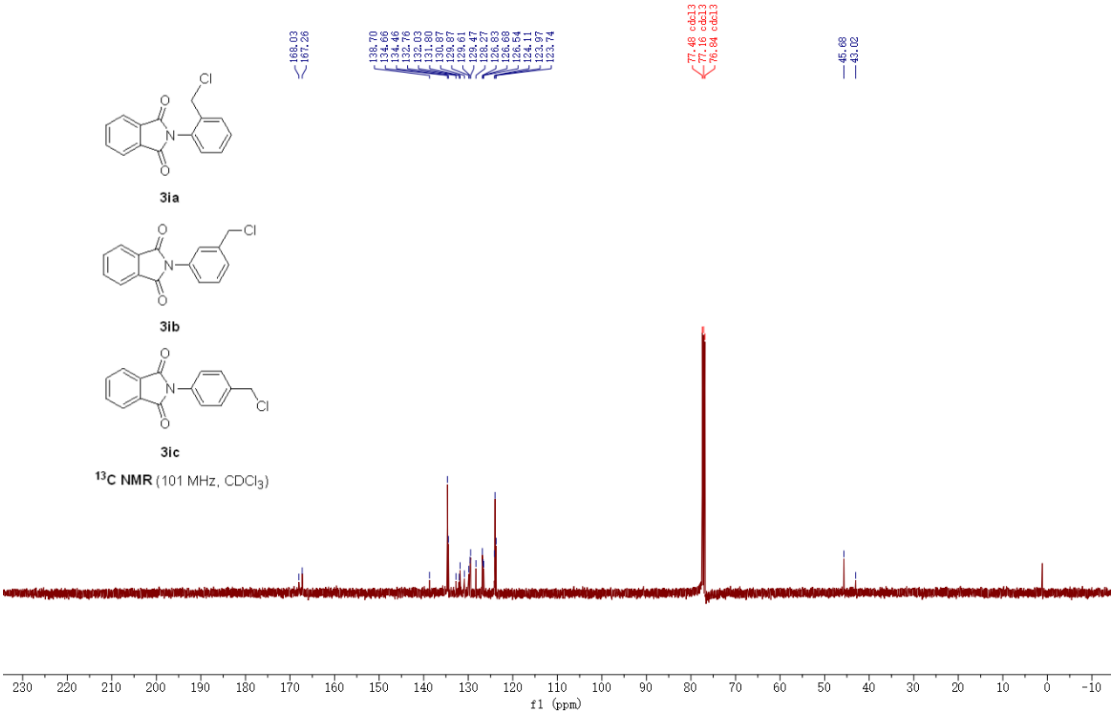
3ia
3ib
3ic
¹³C NMR (101 MHz, CDCl₃)
168.03 167.26
138.70 134.66 134.46 132.76 132.03 131.80 130.87 129.87 129.61 129.47 128.27 126.83 126.68 126.54 124.11 123.97 123.74
77.48 cdcl3 77.16 cdcl3 76.84 cdcl3
45.68 43.02
f1 (ppm)

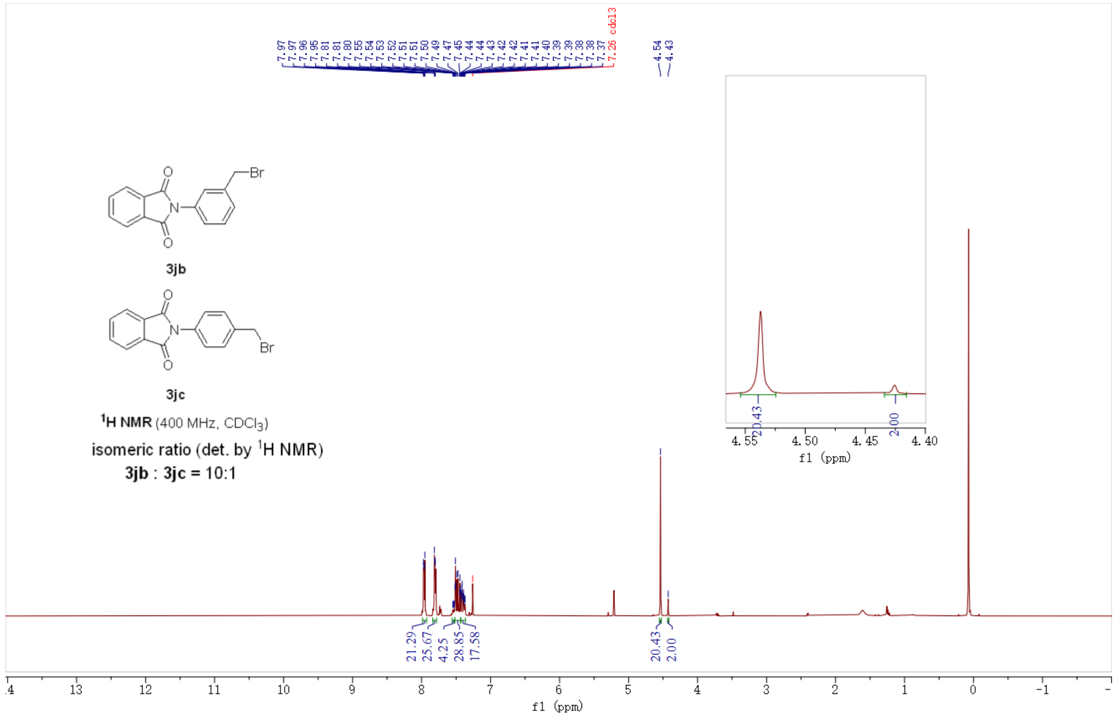
3jb
3jc
1H NMR (400 MHz, CDCl3)
isomeric ratio (det. by 1H NMR)
3jb : 3jc = 10:1
f1 (ppm)

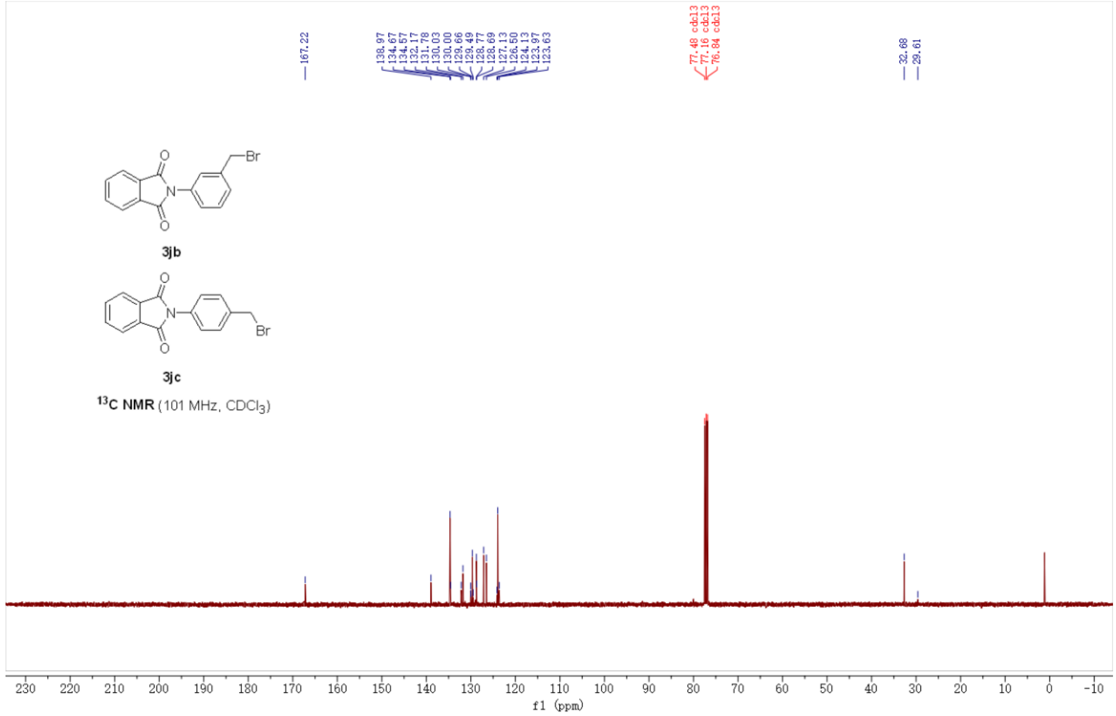
3jb
3jc
13C NMR (101 MHz, CDCl3)
f1 (ppm)

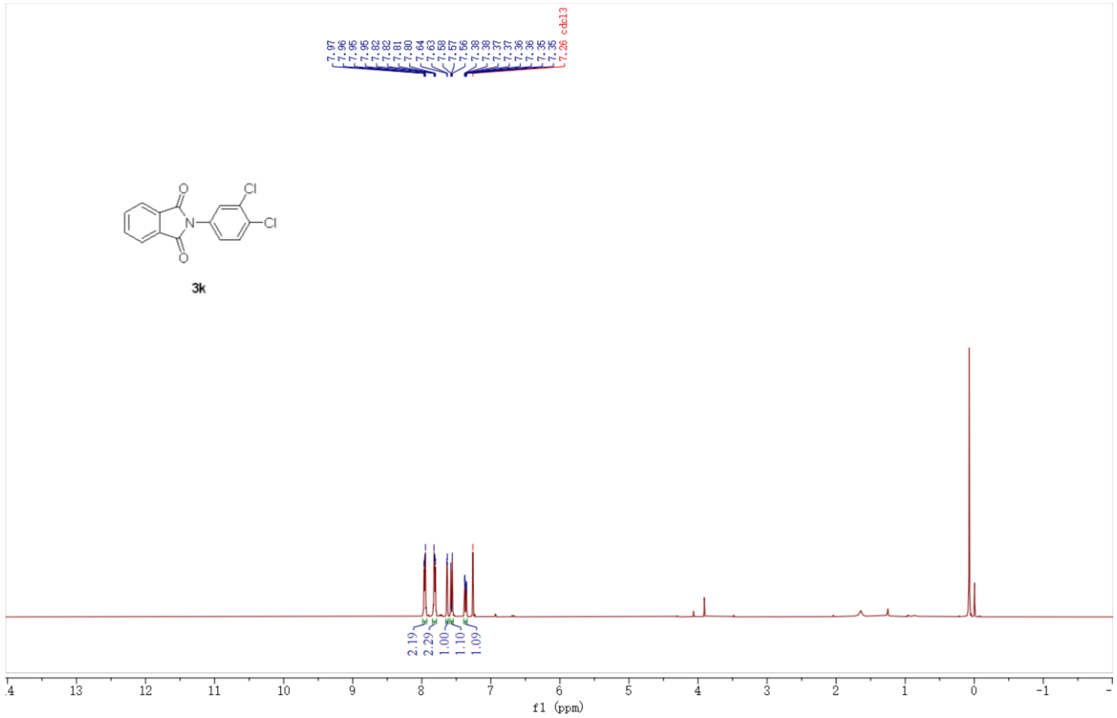
7.97
7.96
7.95
7.95
7.82
7.82
7.81
7.80
7.64
7.63
7.58
7.57
7.56
7.38
7.38
7.37
7.37
7.36
7.36
7.35
7.35
7.26 cdcl3
Cl
Cl
O
N
O
3k
2.19
2.29
1.00
1.10
1.09
f1 (ppm)

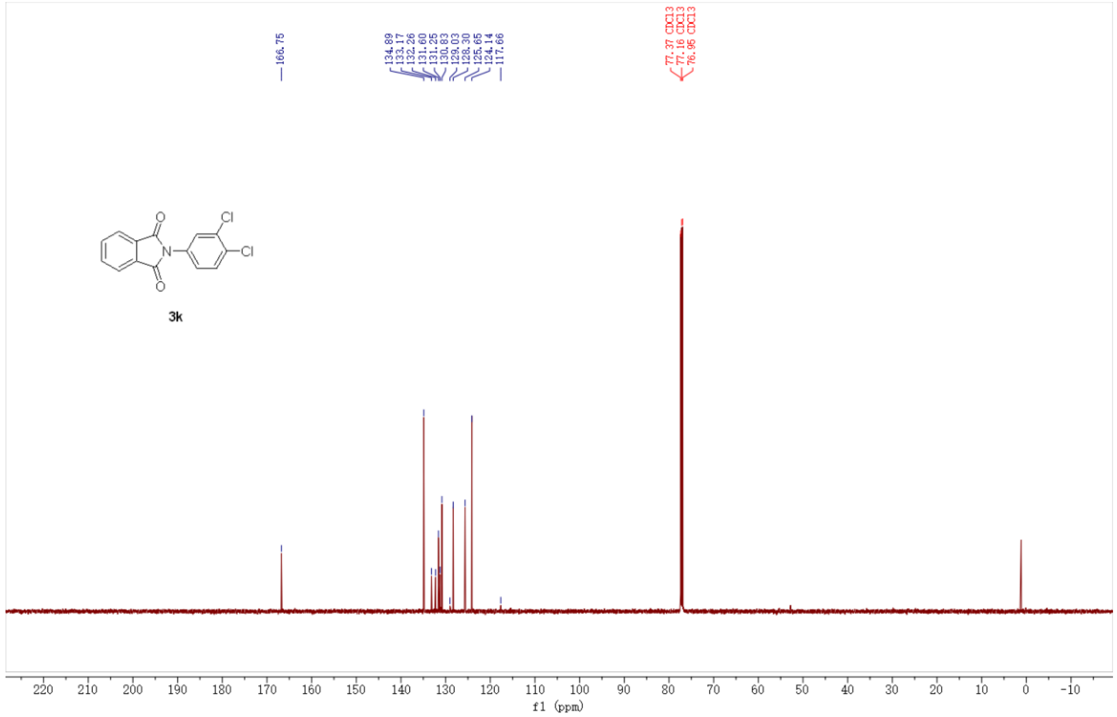
166.75
134.89
133.17
132.26
131.60
131.25
130.83
129.03
128.30
125.65
124.14
117.66
77.37 CDCl3
77.16 CDCl3
76.95 CDCl3
Cl
Cl
O
N
O
3k
f1 (ppm)

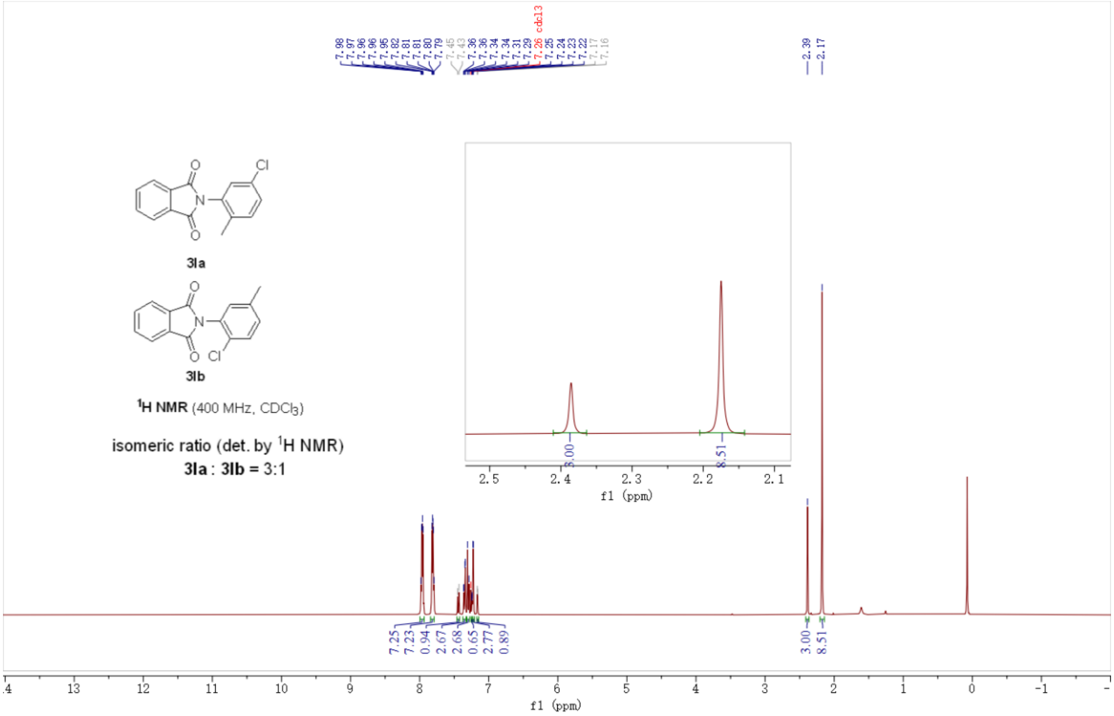
3la
3lb
¹H NMR (400 MHz, CDCl3)
isomeric ratio (det. by ¹H NMR)
3la : 3lb = 3:1
f1 (ppm)

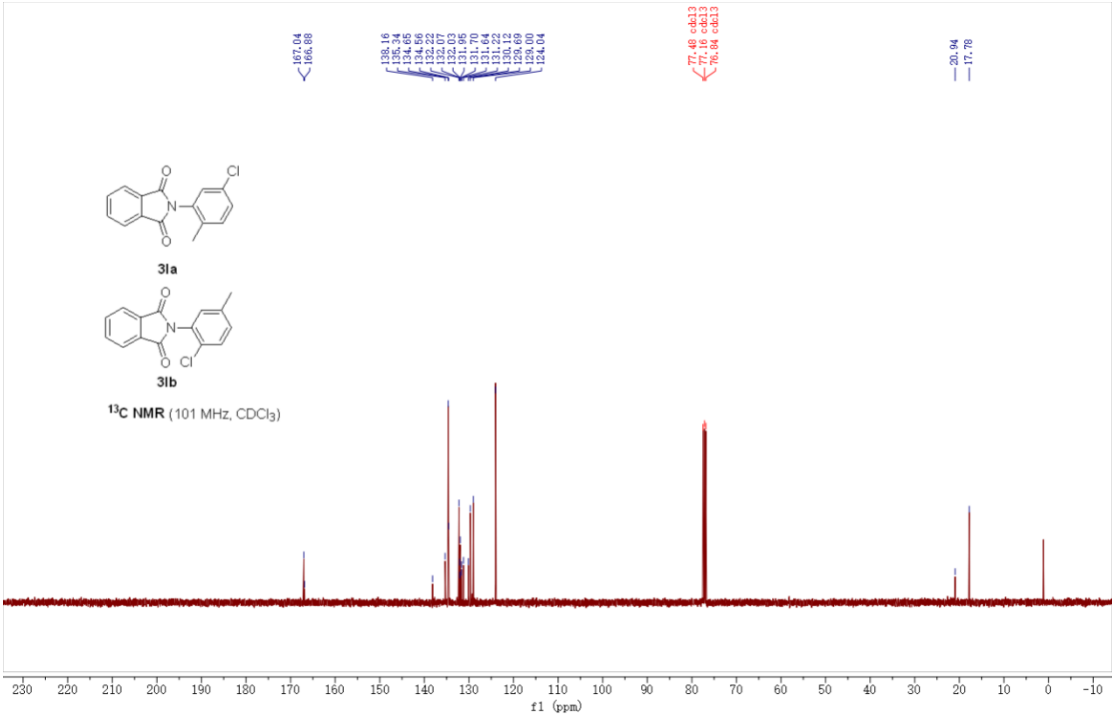
3la
3lb
¹³C NMR (101 MHz, CDCl3)
f1 (ppm)

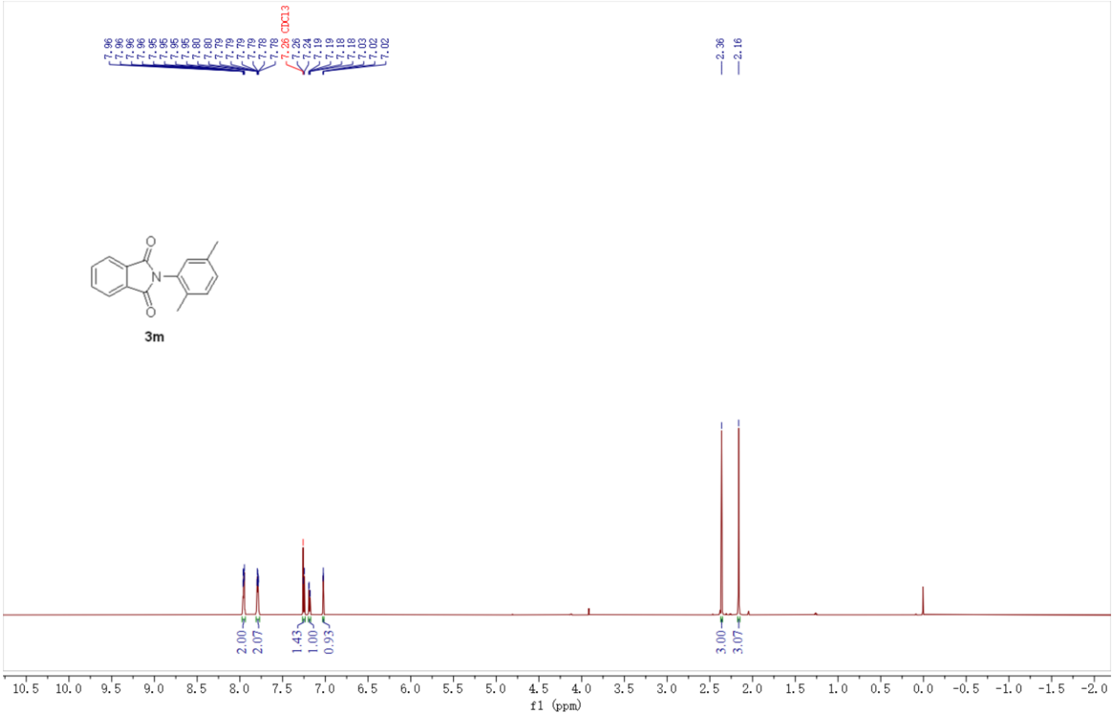

7.96
7.96
7.96
7.96
7.95
7.95
7.95
7.95
7.80
7.80
7.79
7.79
7.79
7.79
7.78
7.28 CDCl3
7.26
7.24
7.19
7.19
7.18
7.18
7.03
7.02
7.02
2.36
2.16
3m
2.00
2.07
1.43
1.00
0.93
3.00
3.07
f1 (ppm)

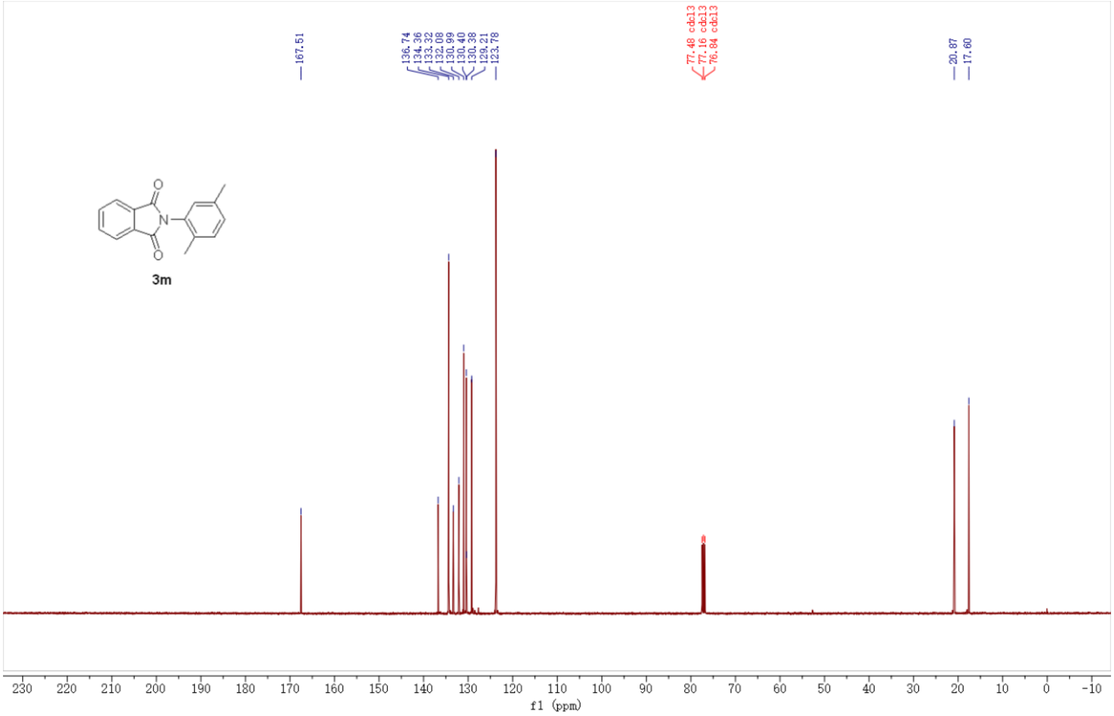

167.51
136.74
134.36
133.32
132.08
130.99
130.40
130.38
129.21
123.78
77.48 cdcl3
77.16 cdcl3
76.84 cdcl3
20.87
17.60
3m
f1 (ppm)

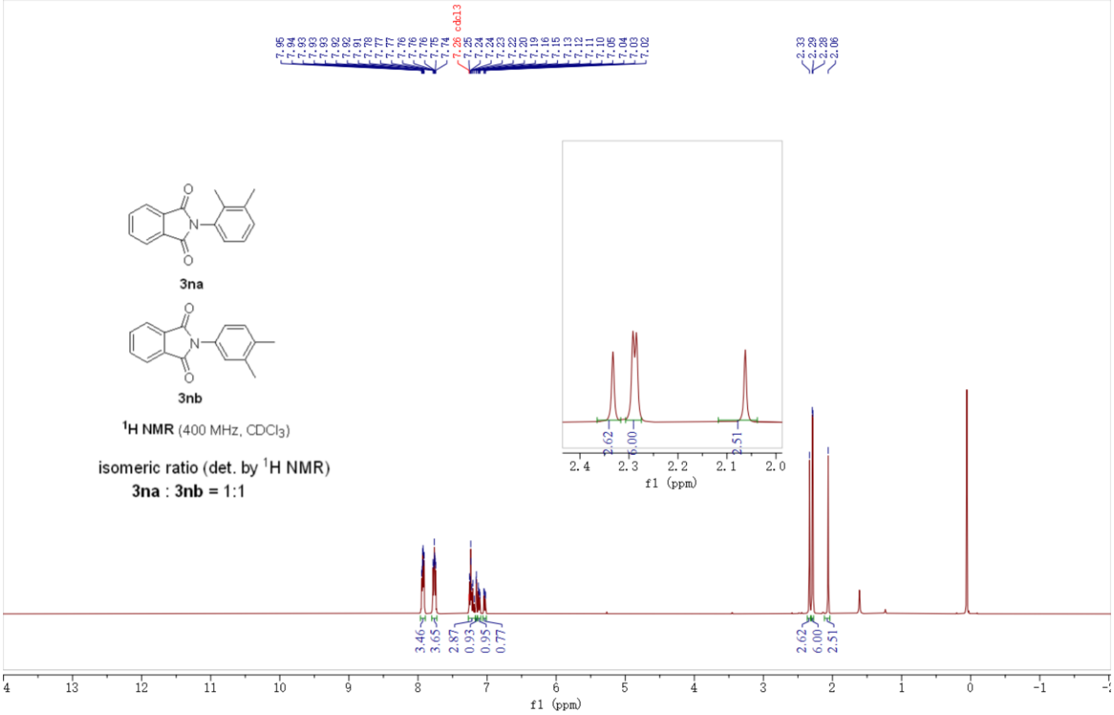
7.95 7.94 7.93 7.93 7.92 7.92 7.91 7.78 7.77 7.77 7.76 7.76 7.75 7.74
7.26 cdcl3
7.25 7.24 7.24 7.23 7.22 7.20 7.19 7.16 7.15 7.13 7.12 7.11 7.10 7.05 7.04 7.03 7.02
2.33 2.29 2.28 2.06
3na
3nb
^{1}H NMR (400 MHz, $CDCl_3$)
isomeric ratio (det. by ^{1}H NMR)
3na : 3nb = 1:1
3.46 3.65 2.87 0.93 0.95 0.77
2.62 6.00 2.51
f1 (ppm)

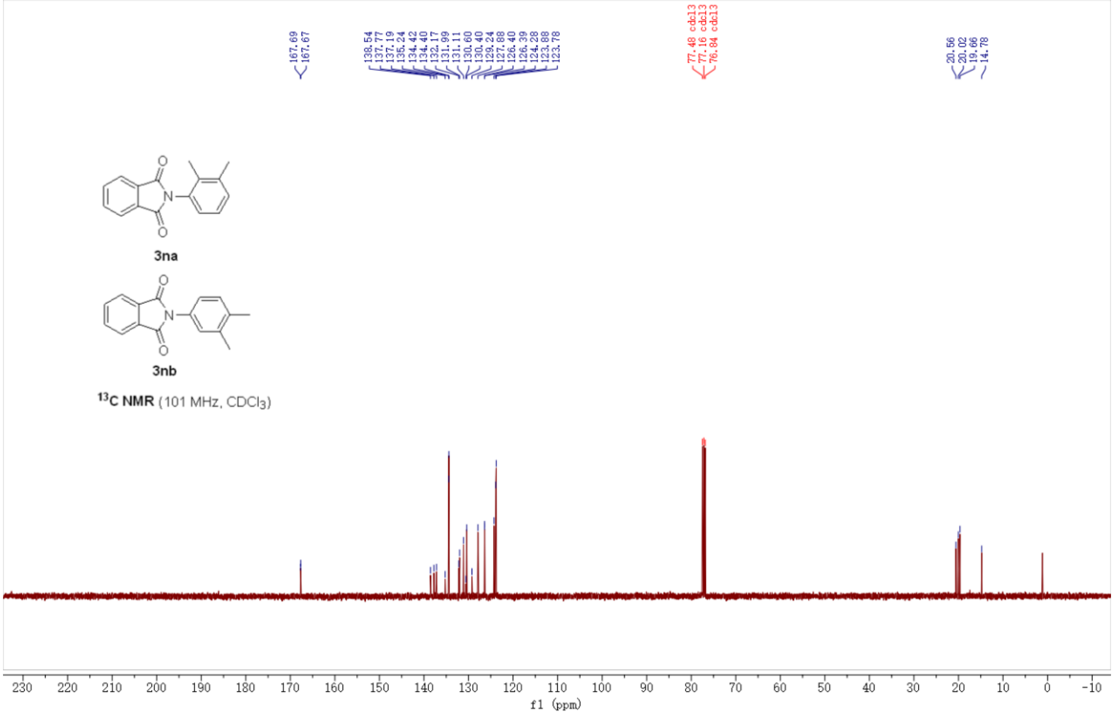
167.69 167.67
138.54 137.77 137.19 135.24 134.42 134.40 132.17 131.99 131.11 130.60 130.40 129.24 127.88 126.40 126.39 124.28 123.88 123.78
77.48 cdcl3 77.16 cdcl3 76.84 cdcl3
20.56 20.02 19.66 14.78
3na
3nb
^{13}C NMR (101 MHz, $CDCl_3$)
f1 (ppm)

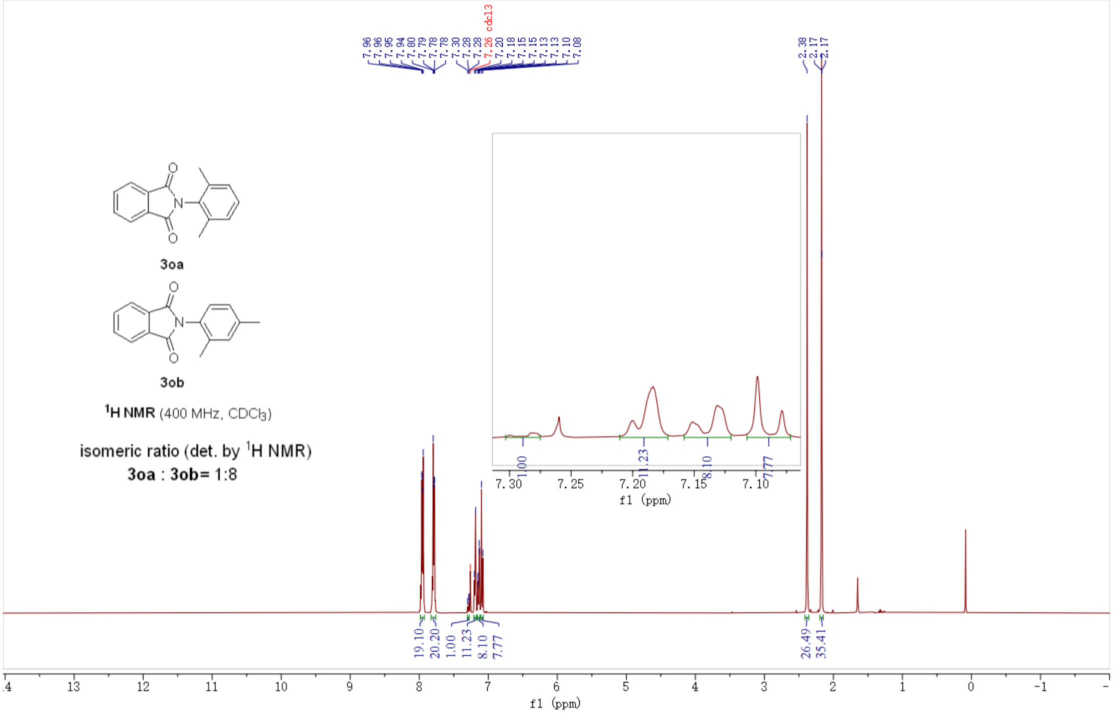
3oa
3ob
1H NMR (400 MHz, CDCl3)
isomeric ratio (det. by 1H NMR)
3oa : 3ob= 1:8
f1 (ppm)

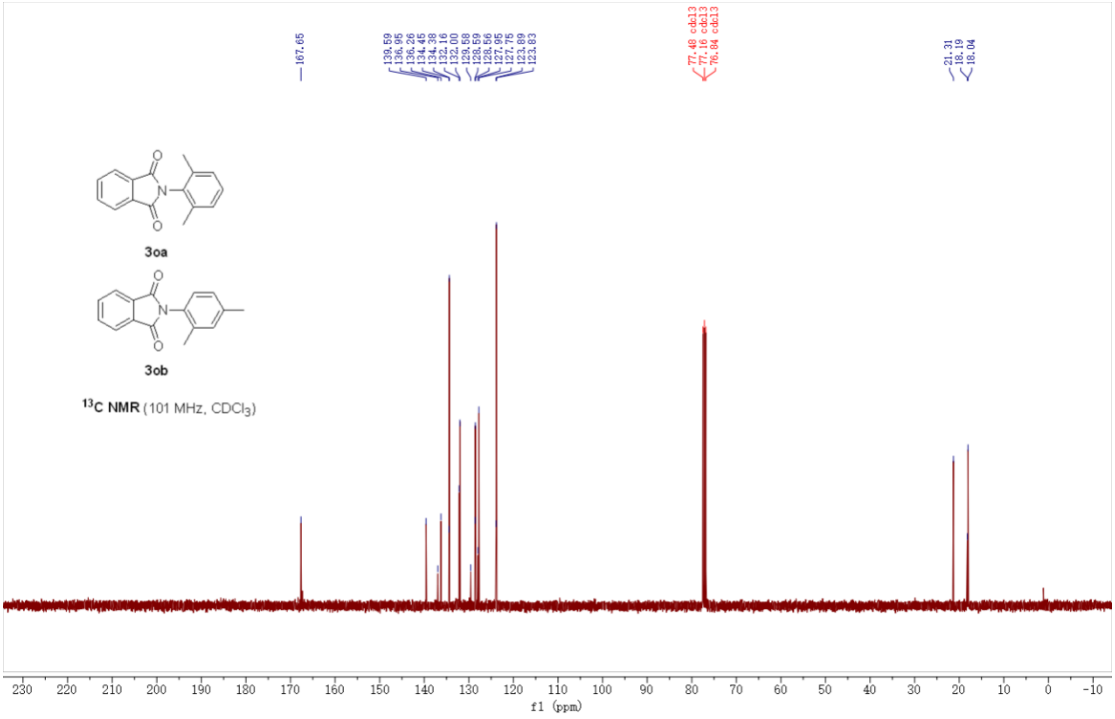
3oa
3ob
13C NMR (101 MHz, CDCl3)
f1 (ppm)

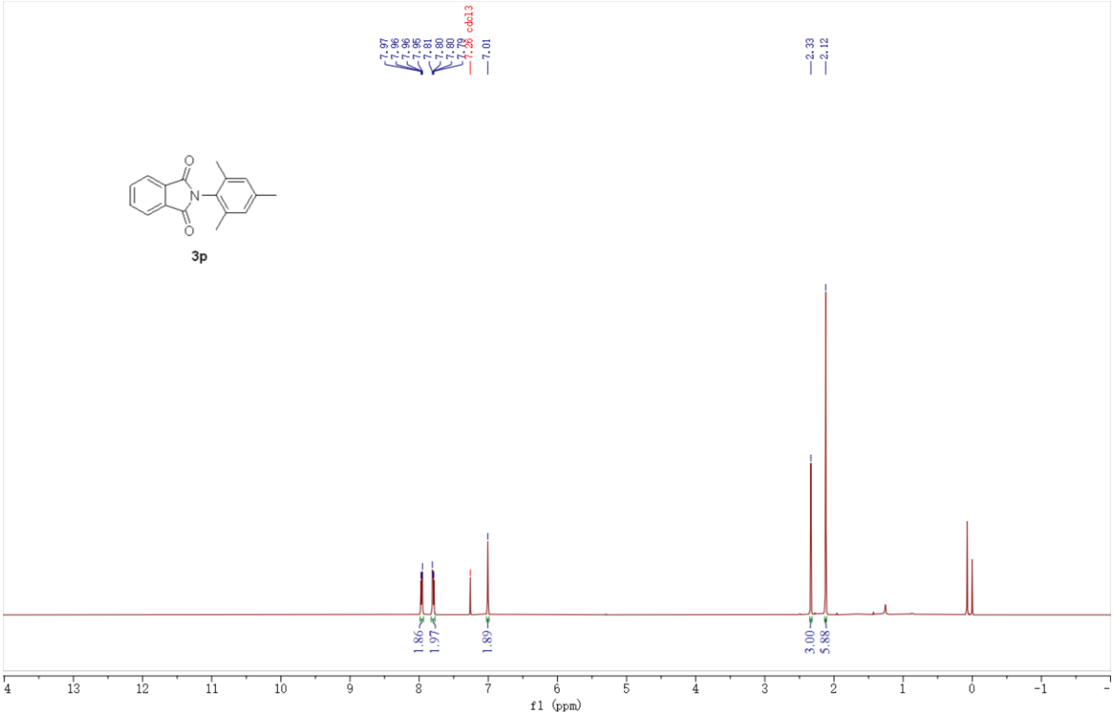
7.97
7.96
7.96
7.95
7.81
7.80
7.80
7.79
7.26 cdcl3
7.01
2.33
2.12
3p
1.86
1.97
1.89
3.00
5.88
f1 (ppm)

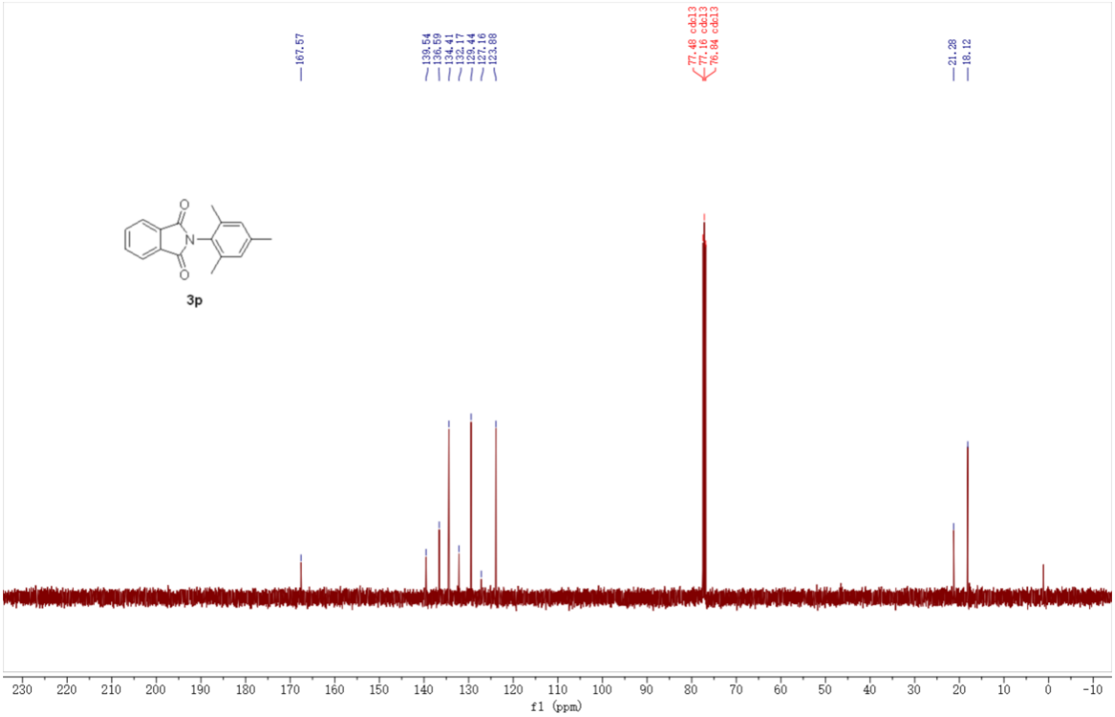
167.57
139.54
136.59
134.41
132.17
129.44
127.16
123.88
77.48 cdcl3
77.16 cdcl3
76.84 cdcl3
21.28
18.12
3p
f1 (ppm)

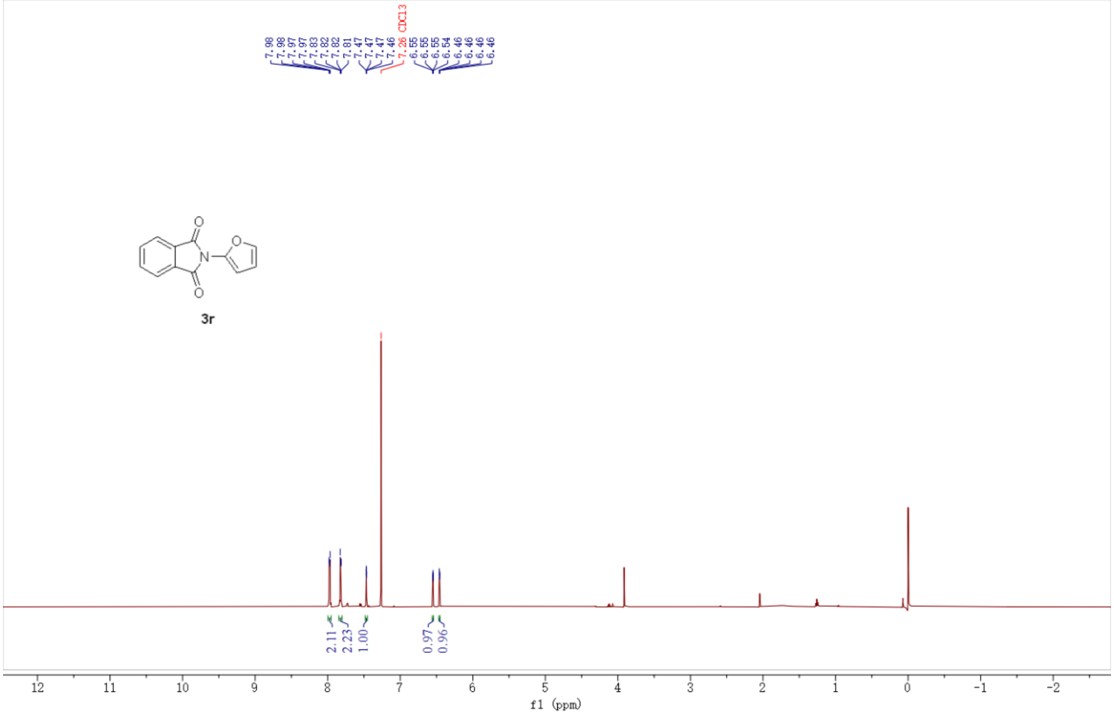
7.98
7.98
7.97
7.97
7.83
7.82
7.82
7.81
7.47
7.47
7.47
7.46
7.26 CDCl3
6.55
6.55
6.55
6.54
6.46
6.46
6.46
6.46
O
N
O
O
3r
2.11
2.23
1.00
0.97
0.96
12
11
10
9
8
7
6
5
4
3
2
1
0
-1
-2
f1 (ppm)

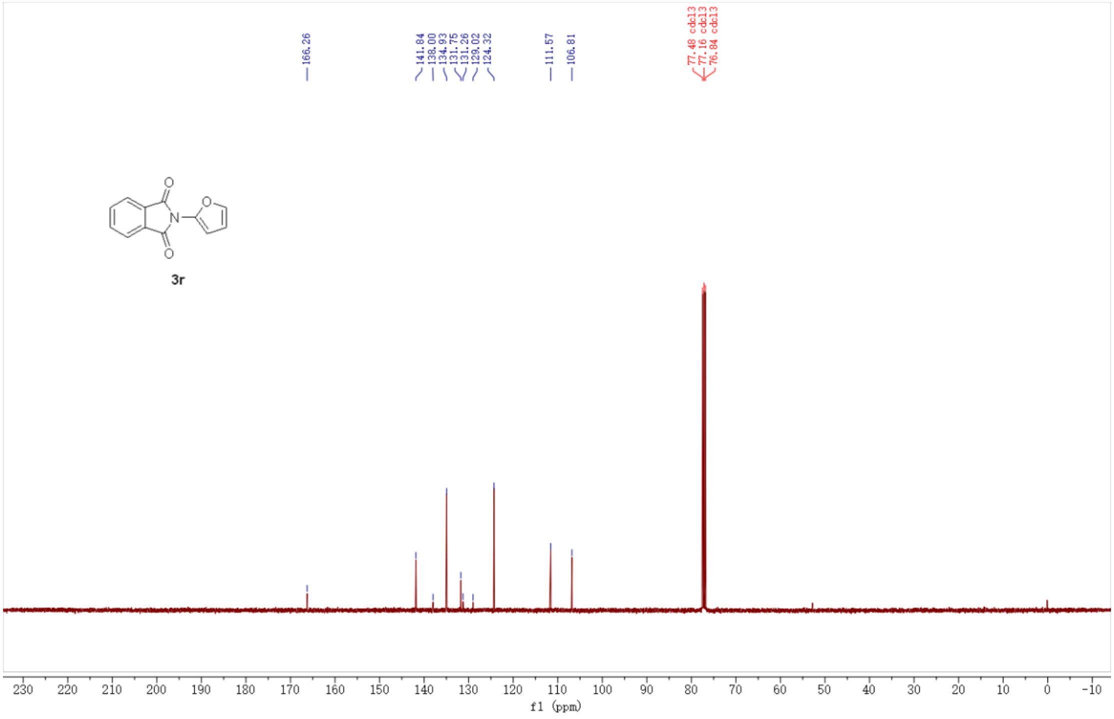
166.26
141.84
138.00
134.93
131.75
131.26
129.02
124.32
111.57
106.81
77.48 cdcl3
77.16 cdcl3
76.84 cdcl3
O
N
O
O
3r
230
220
210
200
190
180
170
160
150
140
130
120
110
100
90
80
70
60
50
40
30
20
10
0
-10
f1 (ppm)

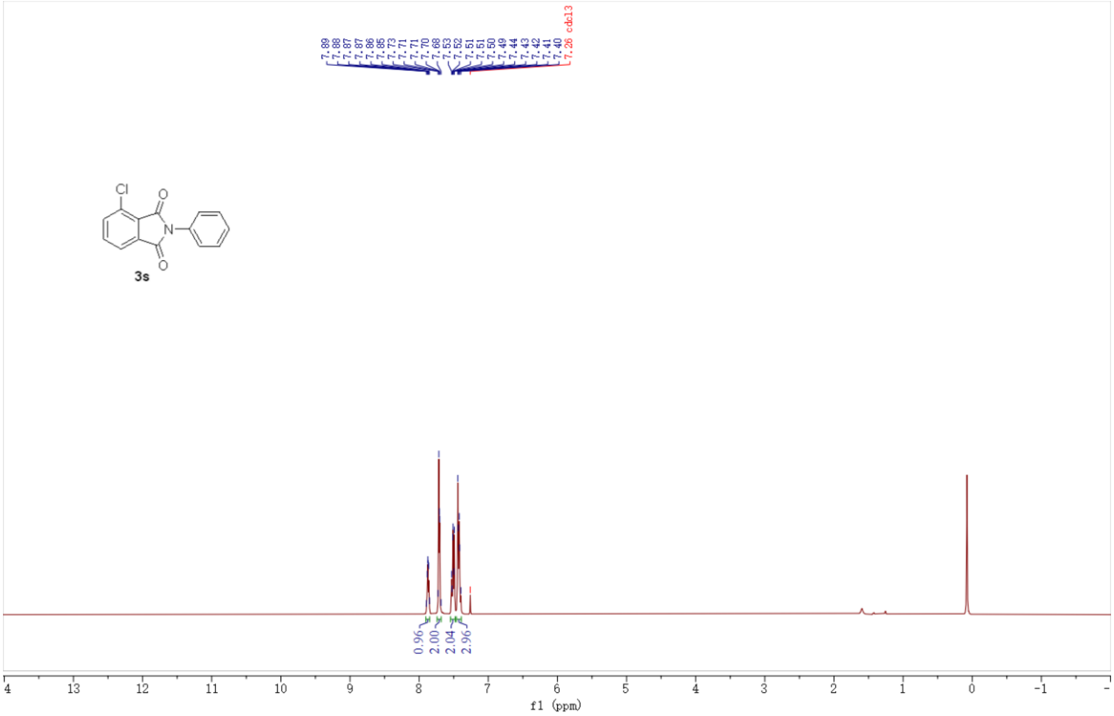
7.89
7.88
7.87
7.87
7.86
7.85
7.73
7.71
7.71
7.70
7.68
7.53
7.52
7.51
7.51
7.50
7.49
7.44
7.43
7.42
7.41
7.40
7.26 cdcl3
Cl
O
N
O
3s
0.96
2.00
2.04
2.96
f1 (ppm)

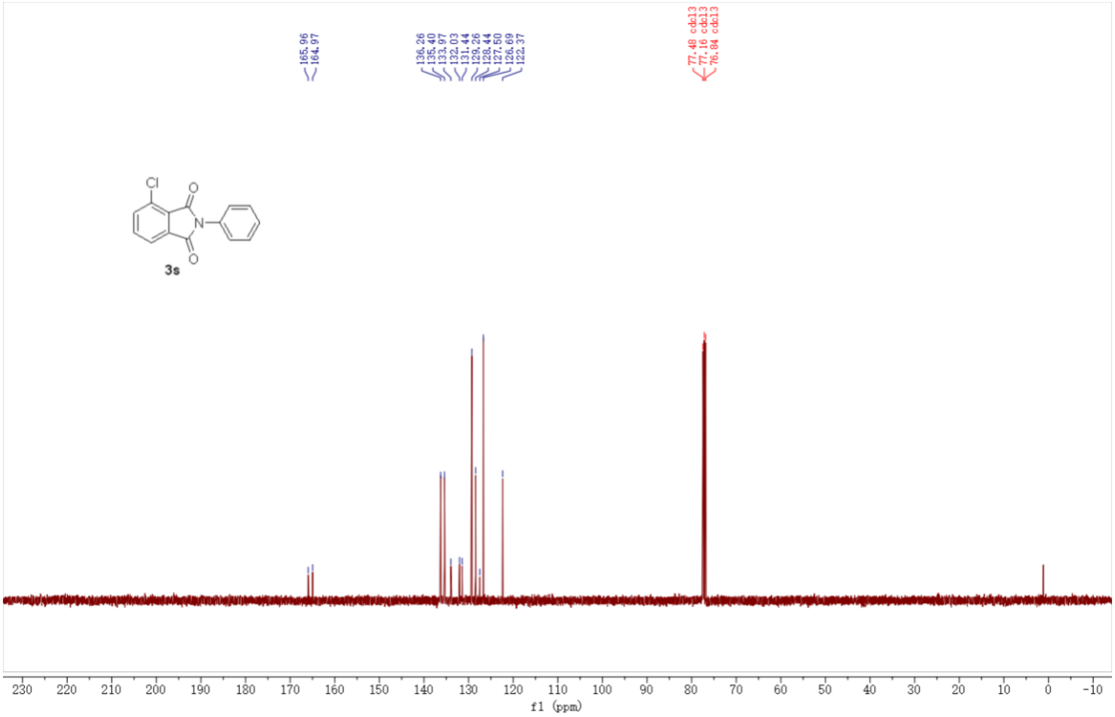
165.96
164.97
136.26
135.40
133.97
132.03
131.44
129.26
128.44
127.50
126.69
122.37
77.48 cdcl3
77.16 cdcl3
76.84 cdcl3
Cl
O
N
O
3s
f1 (ppm)

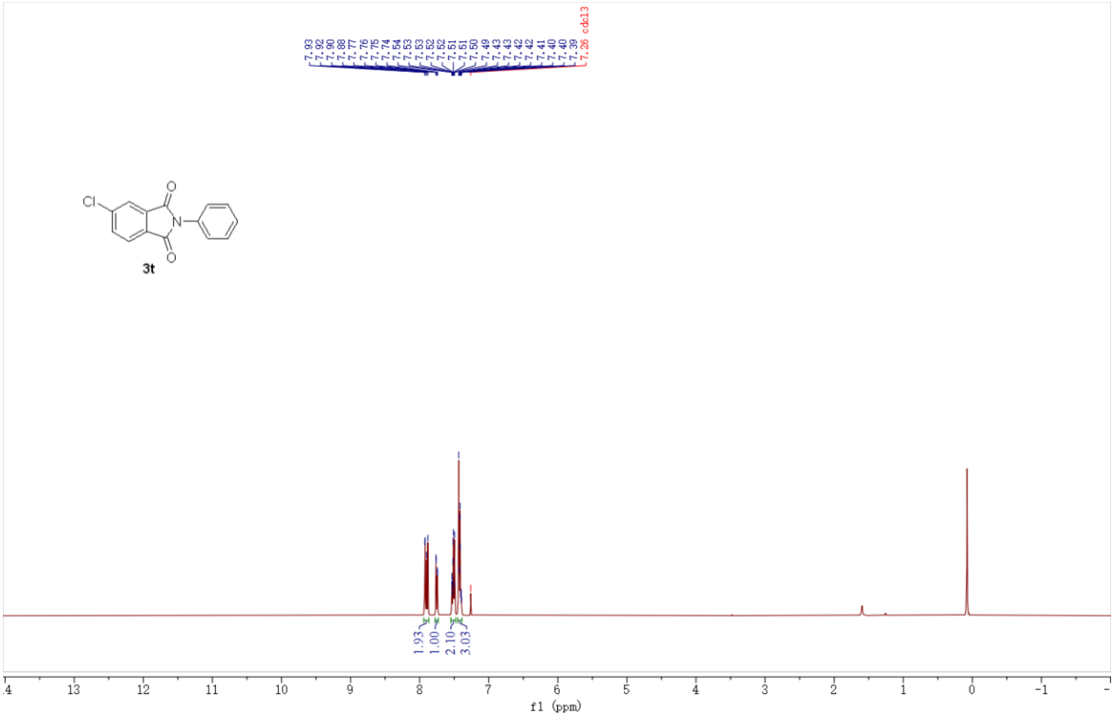

3t
f1 (ppm)

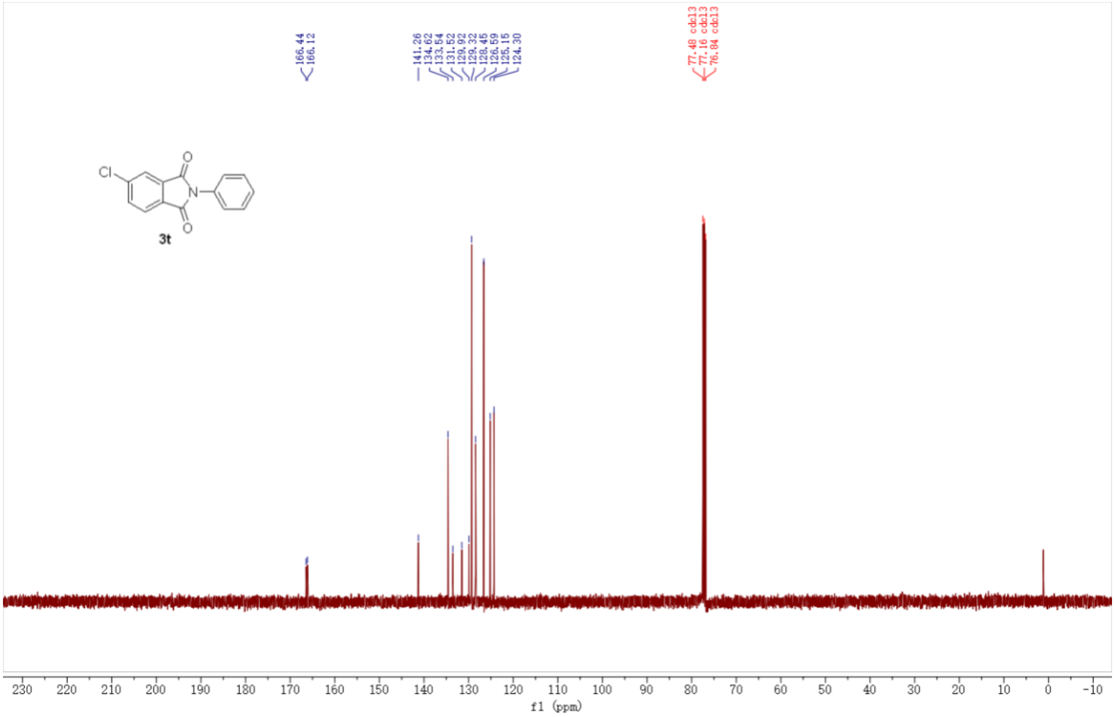

3t
f1 (ppm)

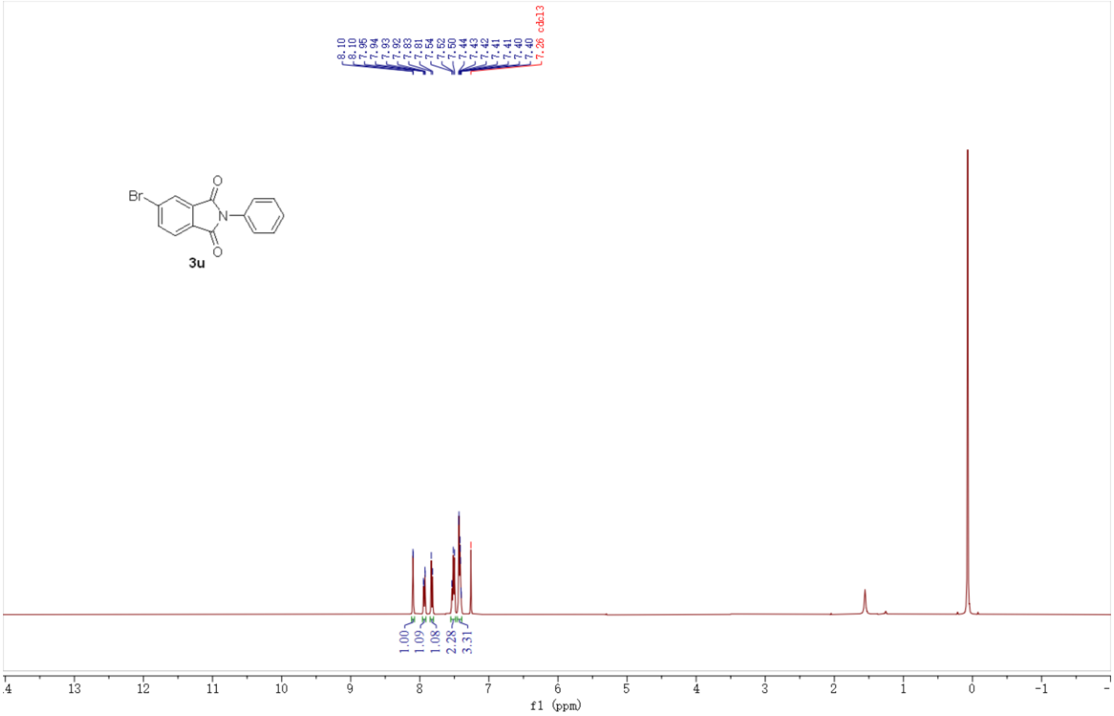

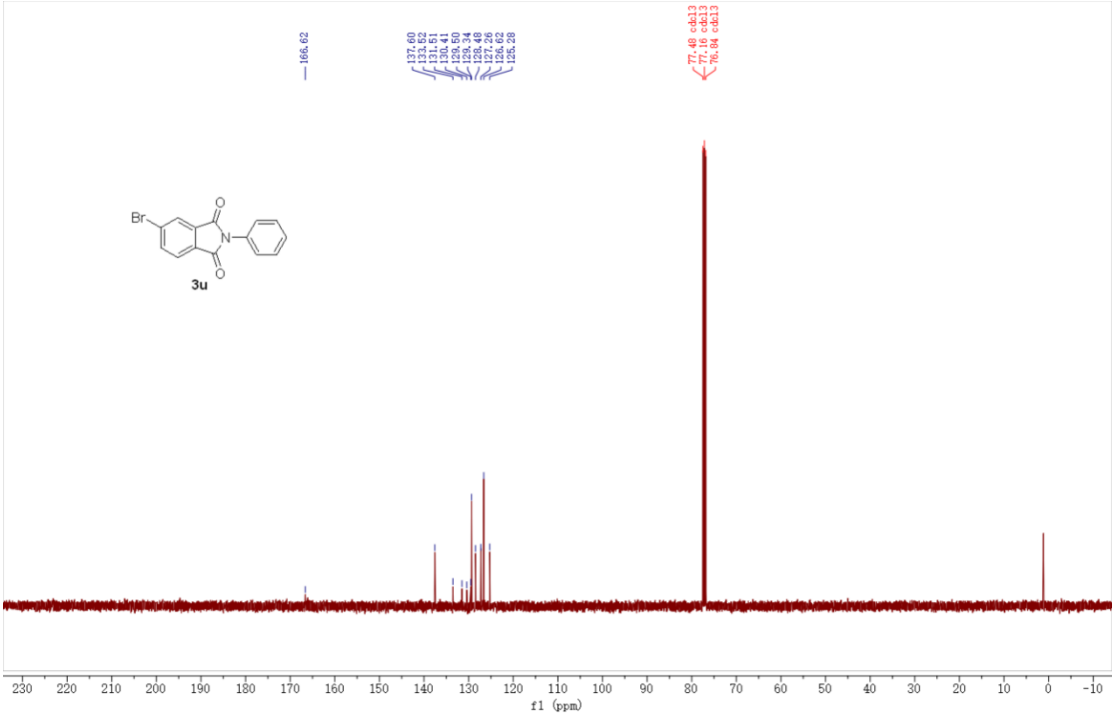

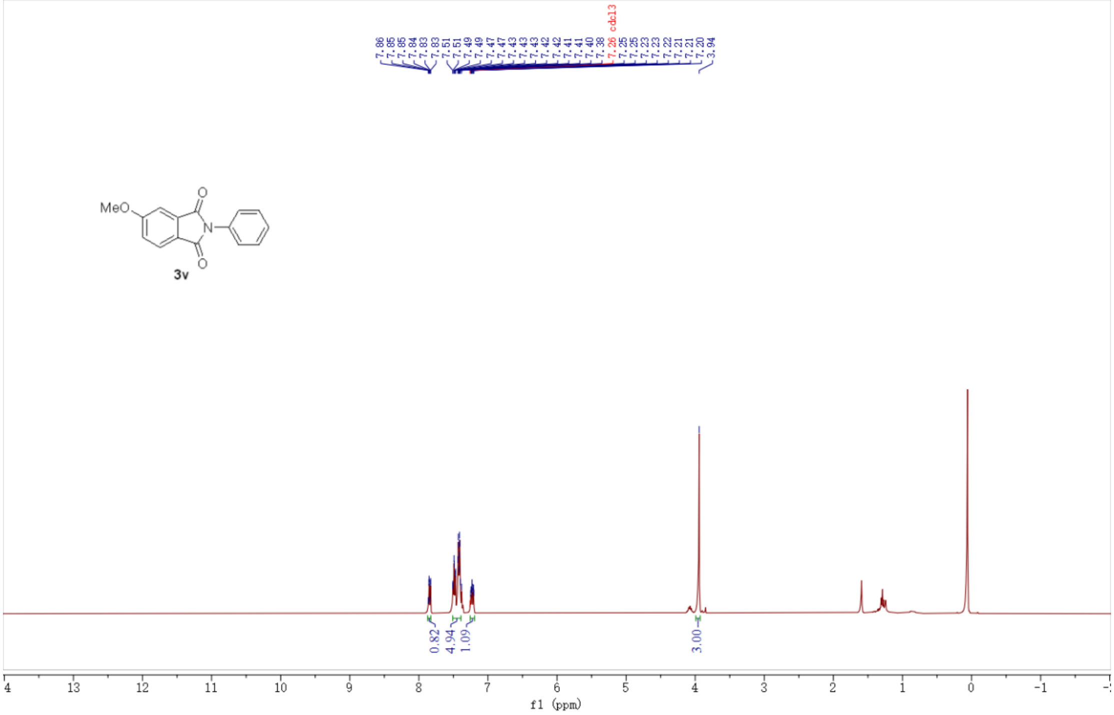
7.86
7.85
7.85
7.84
7.83
7.83
7.51
7.51
7.49
7.49
7.47
7.47
7.43
7.43
7.42
7.42
7.41
7.41
7.40
7.38
7.26 cdcl3
7.25
7.25
7.23
7.23
7.22
7.21
7.21
7.20
3.94
MeO
O
N
O
3v
0.82
4.94
1.09
3.00
f1 (ppm)

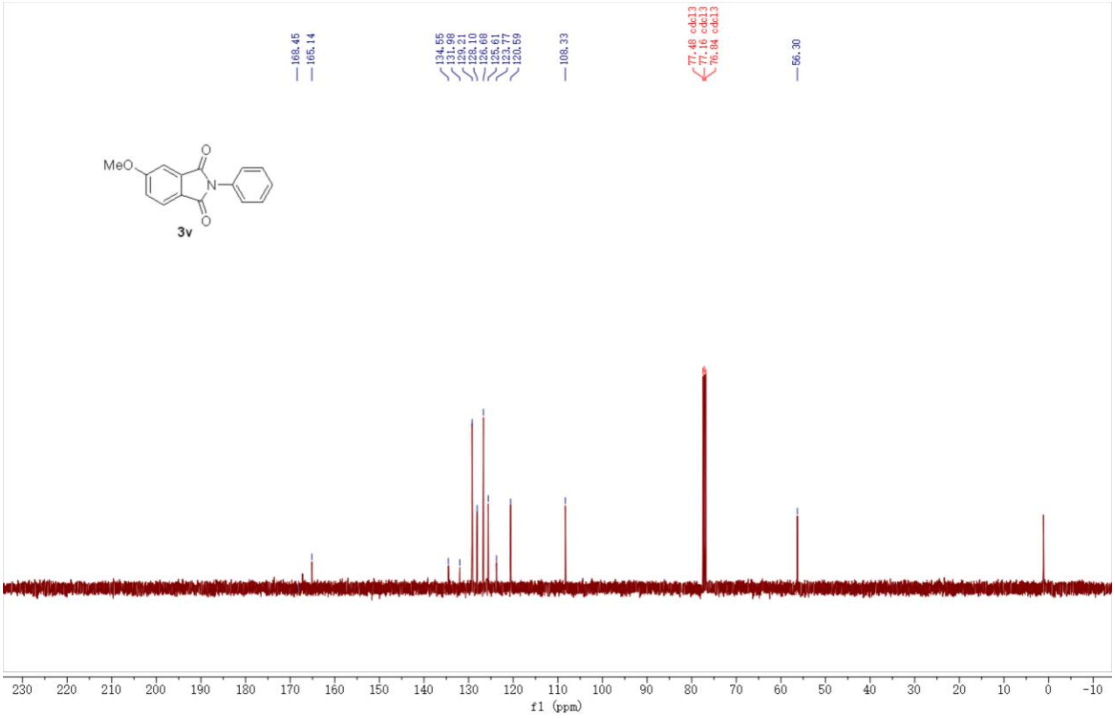
168.45
165.14
134.55
131.98
129.21
128.10
126.68
125.61
123.77
120.59
108.33
77.48 cdcl3
77.16 cdcl3
76.84 cdcl3
56.30
MeO
O
N
O
3v
f1 (ppm)
